# Deep cervical lymphovenous reconstruction in Alzheimer’s disease: three target-defined interfaces with distinct mechanistic implications

**DOI:** 10.3389/fnagi.2026.1945322

**Published:** 2026-09-22

**Authors:** Kuisheng Sun, Youmao Zheng, Li Deng, Wenhua Huang, Wanli Lin

**Affiliations:** 1 Department of Thoracic Surgery, Gaozhou People’s Hospital, Gaozhou, Guangdong, China; 2 Center for Plastic and Reconstructive Surgery, Department of Lymphatic Surgery and Microsurgical Reconstructive Surgery, Zhejiang Provincial People’s Hospital (Affiliated People's Hospital), Hangzhou Medical College, Hangzhou, Zhejiang, China; 3 Guangdong Engineering Research Center for Translation of Medical 3D Printing Application, Guangdong Provincial Key Laboratory of Medical Biomechanics, Department of Human Anatomy, School of Basic Medical Sciences, Southern Medical University, Guangzhou, Guangdong, China

**Keywords:** Alzheimer’s disease, biomarker validation, brain aging, deep cervical lymphatic drainage, glymphatic system, lymphovenous anastomosis, meningeal lymphatics, target engagement

## Abstract

Age-related impairment of brain solute clearance has been implicated in Alzheimer’s disease (AD), yet procedures described as deep cervical lymphovenous reconstruction do not necessarily target the same lymphatic structure. This review examines glymphatic exchange, meningeal lymphatic drainage, cervical outflow, human lymphatic imaging, lymphatic surgery, early clinical studies, registered trials, and reported harms. Three interfaces can be distinguished: lymphatic flap-vein, lymph node-vein, and collecting lymphatic vessel-vein anastomosis. They differ in the reconstructed structure, pressure-flow conditions, sensitivity to nodal resistance and venous back pressure, likely failure modes, and the extent to which patency can be tested directly. Human evidence remains largely uncontrolled, short term, and incompletely reported. Recent mouse, cadaveric, proteomic, and retrospective clinical reports broaden the evidence base but do not establish a human cervical bottleneck or clinical efficacy. Biomarker changes are hypothesis-generating rather than evidence of enhanced cerebral clearance, and a delayed report of neurological, infectious, and immune events indicates the need for surveillance beyond the perioperative period. Credible evaluation requires biological confirmation of AD, a measurable clearance-axis phenotype, a suitable cervical target, explicit operative reporting, directional target-level flow with durable patency, concordant imaging and biomarker findings, perioperative and long-term neurological and systemic safety assessment, a controlled longitudinal design, and blinded patient-level outcomes. Collecting lymphatic vessel-vein anastomosis is the most anatomically defined construct for standardized mechanistic testing; lymph node-vein anastomosis depends on functional nodal screening and sustained flow; and lymphatic flap-vein anastomosis remains an exploratory mixed interface. This is a research-design ordering based on target definition and testability, not a therapeutic ranking, evidence of efficacy, or evidence of clinical superiority.

## 1 Introduction

Alzheimer’s disease (AD) involves accumulation of amyloid beta (Aβ), tau, and inflammation-related products together with failure of several clearance processes. Brain solute export extends beyond the parenchyma through cerebrospinal fluid (CSF)-interstitial fluid (ISF) exchange, meningeal lymphatic drainage, and deep cervical lymphatic outflow. Iliff et al. (2012) demonstrated para-arterial CSF entry and exchange with interstitial solutes, with impaired tracer and Aβ clearance in Aqp4-null mice, although estimates of bulk parenchymal flow remain method-sensitive. Louveau et al. (2015) identified a dural lymphatic network involved in fluid transport and immune trafficking, and Aspelund et al. (2015) showed that dural lymphatics can carry interstitial fluid and macromolecules toward cervical outflow. These studies define a clearance and immune network spanning brain, meninges, and neck, but do not quantify the cervical contribution in humans or identify a surgically correctable bottleneck.

Age affects several components of this pathway at once. In sleeping or anesthetized mice, Xie et al. (2013) reported an approximately 60% expansion of interstitial space with faster metabolite clearance. Hablitz et al. (2020) showed that glymphatic influx and lymphatic efflux follow different circadian phases and that aquaporin-4 (AQP4) contributes to this temporal separation. Da Mesquita et al. (2018) demonstrated age-related meningeal lymphatic dysfunction and found that enhancement mediated by vascular endothelial growth factor C (VEGF-C) improved tracer drainage in aged mice. In humans, Kim et al. (2026) reported differences in deep cervical nodal morphology and microvascular flow in amyloid positron emission tomography (PET)-positive AD. These observations come from animal interventions and cross-sectional human phenotyping rather than a single causal study in people. A distal cervical abnormality may therefore coexist with, or be secondary to, failure elsewhere in the pathway.

Stable-isotope labeling provides direct evidence that Aβ turnover is altered in AD. With approximately 36 h of serial CSF sampling in 12 patients with late-onset AD and 12 age-matched controls, Mawuenyega et al. (2010) found similar Aβ40/42 production but approximately 30% slower clearance in AD. The measure reflects integrated central nervous system (CNS) Aβ turnover and does not identify the fraction carried by meningeal or cervical lymphatics. In aging and AD models, impaired meningeal lymphatic function was associated with greater Aβ burden, reduced CSF-tracer influx, and memory impairment (Da Mesquita et al., 2018). These findings make deep cervical outflow a biologically plausible component of a clearance intervention, but they neither identify the cervical compartment as rate-limiting nor establish clinical efficacy for reconstruction.

Cervical reconstruction is one of several approaches intended to alter brain clearance and should not be presented as a replacement for anti-Aβ or anti-tau treatment. The broader therapeutic landscape includes molecular, pharmacological, physical, lifestyle, and structural interventions (Zhang et al., 2026); an improvement at one compartment may be offset by impaired upstream exchange, downstream resistance, or peripheral handling. Existing appraisals include narrative reviews and a systematic review that searched through July 2025 and identified a small, heterogeneous surgical literature (Lahmar et al., 2026; Chen Q. et al., 2025; Li G. et al., 2026). They highlight unresolved questions about whether brain-derived material reaches a cervical target in measurable amounts, whether that target is rate-limiting, and whether a durable venous bypass increases net clearance without reflux or immune disruption. The distinct contribution of the present review is to separate the lymphatic-side object itself—flap, node, or collecting vessel—and to derive target-specific validation criteria rather than treating deep cervical lymphaticovenous anastomosis (dcLVA) or lymphaticovenous anastomosis (LVA) as one mechanistically uniform intervention.

Procedures described as LVA or dcLVA in AD studies do not necessarily share the same anastomotic target. Three primary lymphatic-side interfaces can be distinguished: a lymphatic flap-vein interface, in which deep cervical lymphatic tissue or a lymph node-adipose flap forms a mixed connection; a lymph node-vein interface, in which a functional node or nodal sinus network is joined to a vein; and a collecting lymphatic vessel-vein interface, in which a defined deep cervical collecting lymphatic lumen is connected to a venous recipient. Primary interface assignment is based on the lymphatic-side structure directly connected to the venous lumen. Local afferent/pre-nodal and efferent/post-nodal relations are described with reference to a specified node, while the primary interface remains defined by the structure connected to the vein (Pan et al., 2010; Murakami et al., 1994; see Section 4.3). On the basis of the operative description and figures, the modified procedure reported by Chen J. Y. et al. (2025) is classified here as a possible mixed lymphatic tissue or lymph node-adipose flap interface. Jin L. et al. (2025) described a related lymph node-adipose flap and external jugular venous bypass; that report is informative for technique and monitoring but does not provide human efficacy evidence.

These targets are not hydrodynamically equivalent. In mice, Yoon et al. (2024) distinguished an upstream nasopharyngeal collecting plexus from downstream valved, smooth-muscle-covered deep cervical collecting vessels. The medial route carried substantially more tracer than a lateral route, aging preferentially affected the upstream plexus, and pharmacological stimulation produced a non-linear response. Browse et al. (1984) showed that a lymph node behaves as a pressure- and flow-sensitive resistance unit rather than as a passive chamber. A shared venous endpoint therefore does not make flap-vein, node-vein, and collecting vessel-vein procedures interchangeable; each creates a different biological and pressure-flow interface.

The main interpretive problem is attribution. A clinical, imaging, or biomarker change cannot be assigned to a cervical operation unless the lymphatic-side target is defined, directional patency persists, patient selection is relevant to age and disease biology, and mechanistic endpoints move coherently. Three questions follow: which interface can be tested most directly, how nodal and venous physiology constrain interpretation, and what evidence would support or refute a role for cervical reconstruction in AD-related clearance biology.

## 2 Review approach and evidence interpretation

The original literature search covered PubMed, Web of Science, and Scopus for English-language peer-reviewed literature published from January 1967 through 15 July 2026. ClinicalTrials.gov, relevant regulatory documents, and backward and forward citation tracking supplemented the database search. Search terms included Alzheimer*, dementia, aging, glymphatic, meningeal lymphatic, deep cervical lymph node, cervical lymphatics, lymphaticovenous anastomosis, lymphovenular anastomosis, lymph node-to-vein, lymphatic flap-vein, lymph node-adipose flap, cervical lymphatic bypass, cerebrospinal fluid drainage, and nasopharyngeal lymphatic plexus. During revision, targeted PubMed searches were performed on 22 August 2026 using combinations of Alzheimer*, dementia, deep cervical, cervical lymphatic, lymphovenous anastomosis, lymphaticovenous, lymphovenular, lymph node-to-vein, lymphatic-venous anastomosis, cervical lymphatic bypass, and dcLVA. Exact-phrase searches, publisher-record checks, and backward and forward citation tracking were used to identify recently published or online-ahead-of-print records not consistently indexed. The update covered records available through 22 August 2026. Web of Science and Scopus were not re-run during revision; the targeted update was not a new systematic search. Screening and interpretation remained centered on the reported or anatomically inferable lymphatic-side interface.

The aim was conceptual classification and mechanistic interpretation rather than effect-size synthesis; the article was therefore conducted as a narrative review, not a systematic review or meta-analysis. Eligible sources included peer-reviewed mechanistic, anatomical, imaging, lymphatic-surgery, biomarker, safety, and human clinical studies, together with registered trials and public regulatory material needed to define the field boundary. Media reports, unpublished claims, and records without a stable scholarly or registry source were not used to support the interface hierarchy. No formal risk-of-bias instrument, Grading of Recommendations Assessment, Development and Evaluation (GRADE) assessment, or quantitative synthesis was applied.

Each source was classified by its contribution to aging-related mechanism, human anatomical measurability, technical feasibility, target engagement, biological response, clinical signal, safety, or trial readiness. Primary interface classification followed the lymphatic-side structure directly connected to the venous lumen. Afferent/pre-nodal and efferent/post-nodal are used as local anatomical descriptors relative to a specified node, not as additional operative categories; a local relationship not established by the source report is left unspecified. Studies that specified the lymphatic-side target, recipient vein, operative configuration, and postoperative patency assessment were given greater interpretive weight. Mechanistic plausibility alone was not treated as clinical efficacy; efficacy would require durable target-level flow linked to biological engagement and blinded patient-level outcomes.

Recent surgical reports were also assessed against a basic verifiability standard. Sources with stable bibliographic or trial identifiers but insufficient target-identification criteria, recipient-vein selection, operative detail, or objective postoperative patency assessment were interpreted only as early signals or clues to terminology. Several case-based and registered sources remain too incompletely described to assign confidently to one of the three interfaces.

Peripheral lymphedema surgery provides technical context but not AD efficacy evidence. In vascularized lymph node transfer, an intact nodal flap is moved with its vascular pedicle, and the arterial and venous pedicle vessels—not the nodal sinus—are anastomosed to recipient vessels. It should therefore not be conflated with lymph node-vein anastomosis (Almadani et al., 2024; Raju and Chang, 2015).

## 3 Aging-related dysfunction of the brain-meningeal-cervical clearance axis

### 3.1 Glymphatic exchange, sleep, and circadian regulation

No single pathway accounts for clearance of Aβ, tau, and inflammation-related macromolecules. CSF-ISF exchange, blood–brain barrier transport, cellular uptake and enzymatic degradation, perivascular drainage, meningeal lymphatics, and cervical outflow operate in parallel. Tarasoff-Conway et al. (2015) emphasized that these routes can compensate for or constrain one another, arguing against a single-outlet model of AD clearance. Ahn et al. (2019) showed that skull-base lymphatics around cranial nerves, vessels, and foramina have structural features suited to CSF uptake and that drainage declines with age in mice. Patel et al. (2019) showed that impaired dural lymphatic drainage delays extracellular tau clearance in mice. These studies support a multi-compartment network, but their animal and method-specific findings do not establish human flow fractions or the effect of cervical surgery.

Figure 1 conceptually depicts the anatomical relation among parenchymal glymphatic exchange, dural meningeal lymphatics, and downstream nasal and cervical outflow; the convective interstitial flux and para-venous efflux in Panel C are proposed pathways rather than directly quantified human transport.

**Figure 1:**
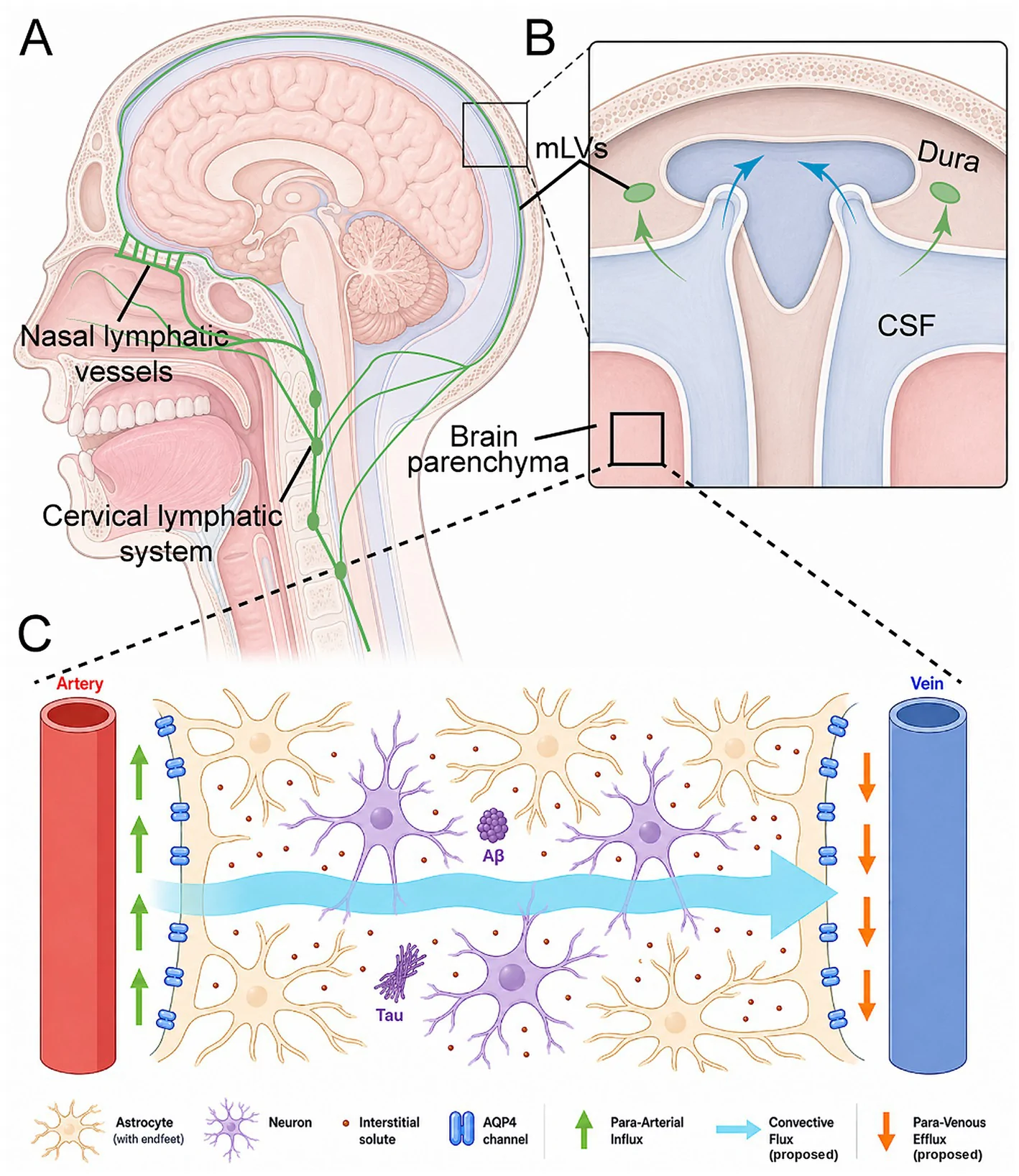
Conceptual anatomical organization of the brain-meningeal-cervical clearance axis in Alzheimer’s disease. **(A)** Sagittal view of CSF-related and lymphatic routes linking the cranial compartment with nasal and cervical pathways. **(B)** Dural sinus region showing meningeal lymphatic vessels within or along the dura and their relation to CSF-derived solute transport from the subarachnoid compartment. **(C)** Conceptual glymphatic transport model showing proposed para-arterial influx, AQP4-associated astrocytic exchange, proposed convective interstitial flux, and proposed para-venous efflux, with Aβ and tau as representative disease-related solutes. The proposed transport arrows are schematic: they do not quantify human flow, establish relative pathway dominance, or demonstrate surgical target engagement. AQP4, aquaporin-4; Aβ, amyloid beta; CSF, cerebrospinal fluid; mLVs, meningeal lymphatic vessels.

Clearance varies with physiological state. Xie et al. (2013) reported an approximately 60% increase in interstitial-space volume during sleep or anesthesia in mice, together with enhanced CSF exchange and faster Aβ removal. In 13 healthy adults, Fultz et al. (2019) observed an ordered sequence during non-rapid-eye-movement sleep in which large slow waves were followed by global blood-oxygen-level-dependent signal reductions and then prominent ventricular CSF inflow; the oscillation was not a direct measure of net solute clearance. In a randomized study of 26 healthy middle-aged men, Ooms et al. (2014) found an approximately 6% overnight decline in CSF Aβ42 after normal sleep that was prevented by total sleep deprivation, whereas Aβ40 and tau did not show the same pattern. Hablitz et al. (2020) demonstrated circadian control of glymphatic influx and lymphatic efflux with different temporal phases. In chronic insomnia, Wen et al. (2025) found lower cerebrovascular reactivity and diffusion tensor image analysis along the perivascular space (DTI-ALPS) values in 55 patients than in 47 controls, with a positive association between the two metrics; both measures remain indirect and cross-sectional. Because these variables can alter postoperative imaging and fluid measurements independently of surgery, dcLVA protocols should standardize sampling time, sleep stage, posture, respiration, sedation, and vascular state when distinguishing an outlet defect from impaired upstream drive.

Age-related impairment is distributed across the pathway rather than confined to one segment. Sleep and circadian disruption can alter CSF-ISF exchange and the timing of glymphatic influx relative to lymphatic efflux (Iliff et al., 2012; Hablitz et al., 2020). Cervical outflow blockade in amyloid precursor protein/presenilin-1 (APP/PS1) transgenic mice was accompanied by loss of perivascular AQP4 polarization within a broader pathological response (Wang et al., 2019), but this does not show that human cervical outflow failure specifically causes AQP4 disorganization. Meningeal lymphatic dysfunction may limit export from the cranial compartment, while altered collecting-vessel or nodal resistance may impair downstream handling. No current human imaging or biomarker measure captures the entire chain or localizes the dominant failure site by itself.

### 3.2 Meningeal lymphatics and deep cervical outflow

Ligation and blockade experiments establish a functional role for cervical drainage. In 5-month-old APP/PS1 mice, 1 month of deep cervical lymphatic ligation aggravated Aβ deposition, neuroinflammation, synaptic-protein loss, AQP4 depolarization, and behavioral abnormalities (Wang et al., 2019). Kinota et al. (2024) used gadolinium-based magnetic resonance imaging (MRI) to show reduced cervical tracer arrival and greater intracranial retention after acute bilateral deep cervical outflow blockade in rats. Papadopoulos et al. (2025) found different driving forces for deep and superficial cervical pathways and redistribution of flow when one route was impaired. These models show that cervical drainage contributes to CSF homeostasis, but near-complete obstruction is not the inverse of adding a venous bypass; injury from blockade does not predict benefit from reconstruction.

Liu et al. (2026) established a side-to-side deep cervical lymph node-external jugular vein (EJV) anastomosis in 10-month-old male APP/PS1 mice and reported increased clearance of cisterna-magna indocyanine green (ICG) signal after surgery. The model supplies a platform for target-engagement experiments, but short-term tracer movement in a male transgenic model does not establish protein clearance, cognitive benefit, long-term patency, immune safety, or human efficacy.

Human data establish anatomy and measurability rather than surgical efficacy. Albayram et al. (2022) used non-invasive MRI to visualize putative meningeal lymphatic networks with cervical continuity, but the signals were not validated pixel-by-pixel against histology and did not directly measure lumen flow, direction, or solute flux. Kim et al. (2026) compared 25 amyloid PET-positive patients with AD and 25 age-matched controls and evaluated 482 deep cervical nodes; a composite of nodal number, morphology, and microvascular signal yielded an exploratory area under the receiver operating characteristic curve (AUC) of 0.81, with 64% sensitivity and 84% specificity. The node-level analysis, small single-center sample, and cross-sectional design preclude use as a diagnostic or surgical-selection threshold. Complementary mouse work identified the nasopharyngeal lymphatic plexus as an upstream hub and downstream collecting vessels as active conduits (Yoon et al., 2024). In aged mice, 20 min of cervical mechanical stimulation approximately doubled tracer outflow and partly corrected an age-related drainage deficit of about 30%, but the effect was short term, non-surgical, and unrelated to AD outcomes (Jin H. et al., 2025). The combined evidence supports a measurable brain-meningeal-cervical route, not clinical efficacy of surgical reconstruction.

Nguyen et al. (2026) used fresh human cadaver dissection, dural and parietal ICG injection, histology, and simulated robotic lymph node-vein anastomosis (LNVA) to map a posterior route toward level II deep cervical nodes and identify adjacent veins. Because active circulation and lymphatic pumping are absent after death and injected dye may follow passive tissue planes, the findings define an anatomical search space and technical hypothesis rather than *in vivo* directional flux, a pressure-flow bottleneck, or a validated patient target.

### 3.3 Human cervical nodal phenotype and axis coupling

Cervical nodes cannot be assumed to be unloaded, low-resistance conduits. In opportunity specimens from 27 patients undergoing cancer-related surgery, Nauen and Troncoso (2022) found Aβ-positive cells in human lymph nodes and more than 40-fold greater enrichment in cervical than inguinal nodes. Al-Diwani et al. (2025) used ultrasound-guided fine-needle aspiration and paired plasma sampling to show enrichment of several neurodegenerative fluid biomarkers in living human cervical nodes. Such enrichment may reflect arrival, retention, cellular processing, slower degradation, local release, or blood contamination rather than effective clearance flux. Tang et al. (2025) detected tau-related signal in abnormal cervical nodes removed during a dementia reconstruction study. These observations identify the cervical node as an accessible biological compartment, but neither prove obstruction nor validate nodal biomarker concentration as a surgical surrogate.

Long-term disruption of the cervical lymphatic chain provides additional, indirect evidence. In a retrospective cohort of 251 adults aged 60 years or older who underwent neck dissection for head-and-neck cancer, Chao et al. (2024) observed nine dementia events over an average of approximately 8.6 years, or about 0.7 events per 100 person-years; bilateral and more extensive dissection was associated with greater risk. The small event count and confounding by cancer, radiotherapy, chemotherapy, vascular injury, nutrition, smoking, infection, and survivorship prevent causal attribution. In experimental work, long-term cervical lymphadenectomy increased tau phosphorylated at threonine 205 (p-tau205), extracellular signal-regulated kinase 1/2-related stress signaling, neuroinflammation, and behavioral abnormalities (Wu C. et al., 2025). Broad removal of the pathway is not the reciprocal of a focal bypass. The findings nevertheless support long-term neurological follow-up after major cervical lymphatic perturbation.

Taken together, current human evidence supports three narrower propositions: CNS solute turnover is altered in AD; cranial-to-cervical continuity can be visualized or sampled; and deep cervical nodes can contain brain-related biomarkers and may differ morphologically in amyloid-positive AD. It does not establish that reduced deep cervical outflow is the dominant cause of impaired clearance, quantify the fraction of Aβ or tau exported through a specific cervical route, demonstrate a pressure-flow bottleneck, or show that a venous bypass increases total net cerebral clearance. The surgical hypothesis is therefore conditional: a candidate would need an independently measurable downstream abnormality that remains functionally coupled to upstream brain clearance and can be reconstructed without clinically important reflux or loss of immune handling.

## 4 Operative target definition and mechanistic interpretability

The anatomical, hydrodynamic, validation-related, and research-design differences among the three interfaces are summarized in Table 1.

**Table 1:** Operative interfaces and mechanistic interpretability.

Dimension	Lymphatic flap-vein interface	Lymph node-vein interface	Collecting lymphatic vessel-vein interface
Main lymphatic object	Deep cervical lymphatic tissue, lymph node-adipose flap, or retained microchannels connected to a jugular venous side-wall opening.	Functional lymph node or nodal sinus network connected to a vein.	Defined collecting lymphatic vessel lumen connected to a venous or venular lumen.
Interface type	Mixed tissue interface; not a standard single-vessel lumen-to-lumen LVA.	Nodal sinus-to-vein interface.	Defined lumen-to-lumen interface.
Distinct biological consequence	May expose a mixed lymphatic/nodal tissue field to venous blood; dominant filtering and immune-processing effects are uncertain.	Modifies a pressure-sensitive immune-filtering organ and may bypass or alter nodal antigen handling.	For diverted lymph, the Figure 4 connection bypasses the illustrated node. Diversion after a specified node instead follows prior nodal transit. Neither relation alone establishes whole-network filtering or immune safety.
Pressure-flow premise	Outflow may depend on small lymphatics, nodal microstructures, or residual channels; the dominant route and net flow are difficult to quantify.	Requires a functional low-resistance node and is affected by lymph flow, venous pressure, and inflammation.	Closest to an active downstream pumping conduit; requires low venous back pressure and reflux control.
Dependence on nodal function	Variable; may include nodal tissue or mixed microchannels, so nodal contribution must be characterized rather than assumed.	Direct and high; requires preserved nodal uptake, perfusion, transnodal flow, and sufficiently low resistance.	Depends on the upstream pathway and reference node. A collector can be efferent from one node and afferent to the next; document the mapped source, direction, and local nodal relation.
Functional patency assessment	Identify the dominant outflow channel and demonstrate repeated directional outflow.	Demonstrate functional nodal uptake, transnodal flow, recipient-vein compatibility, and repeated postoperative patency.	Demonstrate reproducible target identification, source-linked directional flow, and target-level patency at 3, 6, and 12 months; document local nodal relation when visible.
Major potential failure modes	Uncertain dominant channel, thrombosis, fibrosis, reflux, and loss of tissue viability.	High nodal resistance, inflammation or fibrosis, venous reflux, thrombosis, and altered immune handling.	Target misidentification, thrombosis, reflux, valve or pump failure, and inadequate upstream input.
Interpretable signal and boundary	Feasibility and access signal; limited target-specific durable patency evidence.	Nodal-to-venous diversion and nodal-biomarker signals; limited by uncertain nodal resistance and durability.	Lumen-defined reconstruction signal; AD evidence remains non-standardized and lacks durable target-level proof.
Validation before efficacy inference	Identify the dominant flow channel; demonstrate target-level patency; assess reflux, thrombosis, and linked imaging/biomarker endpoints.	Demonstrate functional nodal uptake, transnodal flow, venous-pressure compatibility, and sustained patency.	Demonstrate target reproducibility, source-linked durable directional flow, anti-reflux control, and linkage to blinded outcomes; do not infer global nodal preservation or bypass from the interface name.

Priority refers to target definition, testability, and evidence generation for research design; it does not indicate efficacy, a clinical recommendation, or therapeutic superiority. AD, Alzheimer’s disease; LVA, lymphaticovenous anastomosis.

Reports labeled deep cervical lymphatic-venous anastomosis often do not specify whether the lymphatic-side structure is a flap, a node, or a collecting vessel. The distinction is substantive because the targets differ in anatomy, source of lymphatic input, pressure-flow assumptions, and postoperative patency criteria. Table 2 maps clinical and translational reports to the three interfaces while leaving disclosure-limited sources unclassified (Li X. et al., 2024; Chen J. Y. et al., 2025; Tang et al., 2025; Ma et al., 2025; X. Wu X. et al., 2025; Fu et al., 2026; Hu et al., 2026; Jiang et al., 2026; X. Chen et al., 2026; L. Jin L. et al., 2026; Li Y. et al., 2026; ClinicalTrials.gov, 2026a; ClinicalTrials.gov, 2026b). Jin L. et al. (2025) remain informative for bypass technique and monitoring rather than AD efficacy.

**Table 2:** Translational maturity of representative efficacy, biomarker, safety, and registered studies.

Study	Design/sample	Reported or inferred target	Evidence role and principal limitation
Li X. et al. (2024)	Single case; *n* = 1; 5-week follow-up.	Insufficient detail to determine.	Operability and hypothesis generation only; no counterfactual, reproducible interface, durable patency, or causal inference.
Chen J. Y. et al. (2025)	Prospective single-arm cohort; *n* = 26; 1 month.	Lymphatic flap-vein.	Median MMSE 3 to 5 and ~60% caregiver-reported improvement; non-significant CSF trends and two transient arm-elevation events; no control, blinded outcomes, or durable patency.
Tang et al. (2025)	Prospective single-arm mechanistic cohort; *n* = 28; approximately 1-week cognitive assessment.	Lymph node-vein.	~64.3% had a higher MMSE at 1 week and nodal tau signal was detected; the window is vulnerable to perioperative and practice effects and does not establish durable tau clearance.
Ma et al. (2025)	Retrospective single-center study; *n* = 41; 3 months.	Collecting lymphatic vessel-vein.	Exploratory 50% CDR-SB response definition, >67% with at least one favorable Aβ42/40 or p-tau181 measure, and two mild adverse events; multiple endpoints, no control, and target-level patency gap.
Fu et al. (2026)	Prospective single-arm study; *n* = 139 severe AD; 6 months.	Collecting lymphatic vessel-vein.	Direct lymphatic-vessel-to-EJV anastomoses with partial regional node resection; local nodal relations insufficiently reported. Single-arm clinical/biomarker signals with reported assessor blinding and a descriptive external lecanemab reference, not a randomized within-study comparison. Delirium/sleep disturbance and one bleeding/hematoma reoperation; no durable source-defined patency or causal attribution.
Wu et al. (2025)	Retrospective pilot; 36 source cases, 10 analyzed; 4 short-term responders; 1 month.	Lymphatic flap-vein.	Within-sample PVS thresholds (~5,150 and ~3,630 mm^3^) produced AUCs of 1.00 and 0.88; severe overfitting risk, no external validation, and no durable patency proof.
Hu et al. (2026)	Exploratory single-arm biomarker study; *n* = 30; 180 days.	Insufficient detail to determine.	Plasma Aβ42 plus Aβ42/40 model AUC 0.737; no control, external validation, target-level patency, source attribution, or clinical-surrogate validation.
Jiang et al. (2026)	Case report; *n* = 1; hemorrhage at 23 days and later seizure/immune findings.	Insufficient detail to determine.	High-importance delayed neurological/infectious/immune safety signal; incomplete operative detail and temporal association cannot establish incidence or causality.
Chen X. et al. (2026)	Retrospective case series; *n* = 5; short follow-up.	Collecting vessel-interposed vein graft-EJV (dynamic neck LVA).	All grafts were described as patent with no recorded postoperative complications; small mean cognitive changes and heterogeneous PET signals. Very small uncontrolled sample, multiple endpoints, measurement sensitivity, and no validation of the proposed dynamic mechanism or long-term safety.
Jin L. et al. (2026)	Retrospective comparative cohort; 6-month lecanemab versus dcLVA; group sizes were not stated in the PubMed abstract.	Insufficient operative detail to classify confidently.	Cognitive decline was reported in 23.5% versus 81.8%, and change in centiloid favored lecanemab; non-randomized treatment selection, unclear interface and patency, and internally derived imaging threshold prevent a causal comparative-effect estimate.
Li Y. et al. (2026)	Preoperative CSF proteomics; *n* = 90; internally defined responders and non-responders.	Interface not reported sufficiently for target classification.	NPTX2/IGSF10 AUC 0.854 and four-marker AUC 0.880; internally derived prediction without external validation, untreated comparison, or target-specific patency. Candidate stratification evidence only.
ClinicalTrials.gov (2026a)	Planned multicenter open-label, blinded-endpoint randomized study; approximately *n* = 376; 12-month core outcomes; no results.	Broadly described deep cervical lymphatic tissue-to-vein procedure.	Important randomized design and multimodal endpoint plan; target-level method, learning curve, patency, harms, and outcomes remain unavailable.
ClinicalTrials.gov (2026b)	Planned multicenter prospective registry; approximately *n* = 814 across ~40 centers; follow-up to 24 months; no results.	Insufficient detail to determine.	Can characterize heterogeneity, learning, rare harms, and external validity; no non-surgical control and therefore not a substitute for randomized evidence.

Classification follows the available titles, abstracts, methods, operative descriptions, figures, and full-text detail. Primary interface assignment is based on the lymphatic-side structure directly connected to the venous lumen. Afferent/pre-nodal and efferent/post-nodal are treated only as local relations to a specified node, not as separate operative categories. Sources with insufficient target disclosure remain unclassified. Newly added reports were interpreted according to the verified information level available; abstract-level comparative results were not used for unreported procedural or causal claims. Temporal association in a case report does not establish causality. Registrations describe planned, not completed, evidence. AD, Alzheimer’s disease; Aβ, amyloid beta; AUC, area under the receiver operating characteristic curve; CDR-SB, Clinical Dementia Rating-Sum of Boxes; CSF, cerebrospinal fluid; dcLVA, deep cervical lymphaticovenous anastomosis; EJV, external jugular vein; IGSF10, immunoglobulin superfamily member 10; LVA, lymphaticovenous anastomosis; MMSE, Mini-Mental State Examination; NPTX2, neuronal pentraxin-2; PET, positron emission tomography; p-tau, phosphorylated tau (numeric suffix denotes the residue); PVS, perivascular space.

Pressure-flow behavior differs sharply among targets. Collecting lymphatic vessels are divided by valves into actively contracting lymphangions, and effective pumping depends on preload, venous afterload, valve competence, and avoidance of sustained reverse flow or inflammatory remodeling (Scallan et al., 2016; Angeli and Lim, 2023). In dogs, Browse et al. (1984) measured mean nodal resistance of approximately 180 mmHg/mL/min below 0.1 mL/min and about 68 above 1.0 mL/min; a 10-mmHg increase in venous pressure raised nodal resistance by approximately 8.6 units, especially at low flow. These values are not human cervical thresholds, but they illustrate why a low-flow node-vein interface may be vulnerable to venous back pressure. In an *in situ* rabbit hind-limb node model, Nagai et al. (2008) found that perfusion pressure, downstream pressure, and acute inflammation altered lymph outflow. The three interfaces therefore differ in their principal resistance source, susceptibility to reflux, immune consequences, and requirements for patency assessment.

Age can reduce physiological reserve on both sides of a lymphovenous interface. Experimental collector studies show loss of smooth-muscle investment and reduced contraction frequency, systolic lymph-flow velocity, and pumping activity with aging (Zolla et al., 2015). Human superficial lymph nodes show age-related lymphocyte loss, fibrosis, and lipomatosis (Hadamitzky et al., 2010), although these findings are not direct measurements of deep cervical nodes in AD. In 349 healthy participants, advancing age was associated with slower internal-jugular venous velocity, altered drainage distribution, and more frequent left jugular venous reflux (Chung et al., 2010). Direct age-specific measurements of human deep cervical lymphatic valve competence are lacking. These cross-compartment data imply reduced reserve and greater sensitivity to venous afterload, but they do not define an age threshold or predict durability of any interface.

### 4.1 Lymphatic flap-vein interface

Lymphatic flap-vein anastomosis denotes a procedure in which deep cervical lymphatic tissue, a lymph node-adipose flap, or another lymphatic tissue-bearing field forms the lymphatic side of the connection. Two current AD studies are mapped to this category (Chen J. Y. et al., 2025; Wu X. et al., 2025). The classification follows the reported lymphatic-side structure directly connected to the vein and does not imply standardized technique, durable patency, or efficacy. Figure 2 depicts the interface.

**Figure 2:**
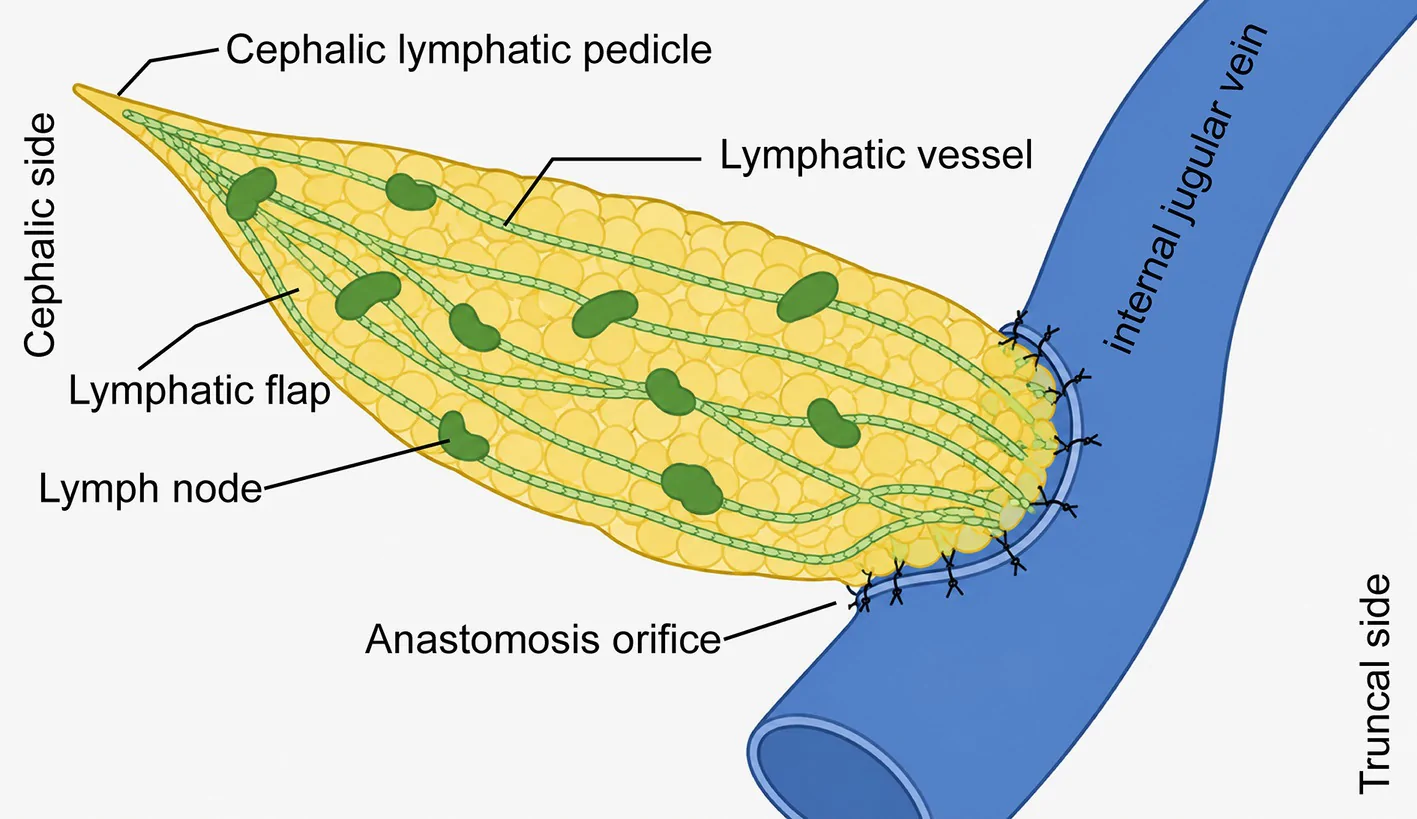
Lymphatic flap-vein interface. Deep cervical lymphatic tissue or a lymph node-adipose flap containing small lymphatic channels and node-like structures is sutured to a venous side-wall opening. The result is a mixed tissue connection rather than a standardized single collecting lymphatic vessel-to-vein anastomosis.

The operation connects a lymphatic tissue field or lymph node-adipose flap to a venous side-wall opening under tracer or near-infrared guidance. It is not a pure collecting-vessel lumen-to-lumen anastomosis. The dominant flow channel, target-level patency, net outflow, reflux, thrombosis, and fibrosis remain unresolved.

Anatomical accessibility does not establish physiologic specificity. In 12 cadavers and 24 neck sides, Yağmurlu et al. (2020) described a mean of approximately 6.9 deep cervical nodes per side and podoplanin-positive channels near the jugular foramen and internal jugular chain, including possible intrajugular lymphatic vessels. These findings define an anatomical search space but do not identify the structure carrying brain-derived fluid *in vivo*. Pan et al. (2010) showed that head-and-neck nodes and collecting vessels receive mixed input from scalp, face, nasal, oral, and pharyngeal territories. Finding lymphatic or nodal tissue near the internal jugular vein is therefore not equivalent to identifying a functional brain-draining collecting vessel suitable for lumen-to-lumen anastomosis.

Peripheral lymphatic surgery offers technical analogies, not AD evidence. Yamamoto and Sugihara (1998) described implantation of multiple small lymphatics into a larger venous lumen to address caliber mismatch, a construct that still depends on preserved upstream flow, low venous pressure, and resistance to thrombosis and fibrosis. In 37 patients with severe upper-extremity lymphosclerosis, Yamamoto et al. (2023) created 98 dermal-adipose lymphatic flap shunts and reported improvements in quality of life, edema index, and cellulitis frequency; the uncontrolled peripheral setting and concomitant care prevent direct transfer to AD. In a controlled rat comparison, Ishiura et al. (2017) observed one-week patency in all 6 standard lymphaticovenular anastomoses but only 2 of 6 lymphovenous implantations, despite intraoperative patency in all procedures. Immediate fluorescence cannot therefore substitute for durable target-level flow, and a mixed tissue interface should not be assumed equivalent to intima-to-intima reconstruction.

These limitations place lymphatic flap-vein anastomosis in an exploratory category for AD research. It can be studied when collecting vessels cannot be identified reproducibly or when deep cervical supermicrosurgical vessel-to-vein reconstruction is not yet standardized. Current reports provide evidence of access, feasibility, intraoperative fluorescence, and possible effect modification by perivascular space (PVS) burden, but not of the dominant flow channel, durable target-specific patency, brain-derived material flux, or clinical efficacy (Chen J. Y. et al., 2025; Wu X. et al., 2025). A mixed tissue-vein connection may also alter nodal filtering or immune handling, which current AD reports have not quantified. Its value lies in access to a lymphatic-rich field; it does not demonstrate that a defined physiological conduit has been reconstructed.

### 4.2 Lymph node-vein interface

LNVA joins an incised nodal sinus network to a vein. Success depends on a node that remains perfused, connected to upstream lymph flow, and sufficiently low resistance—not merely on the larger surface area of the nodal interface. In the canine experiments of Calnan et al. (1967), early function at approximately 2 weeks was lost by two to 3 months under the study conditions, with endothelial overgrowth and intimal-like fibrosis. The model does not establish inevitable failure of modern cervical LNVA, but it separates immediate function from durable flow. Bailey et al. (2024) emphasized preoperative imaging, node quality, recipient-vein selection, technical refinement, and operator experience. In 160 patients with lower-extremity lymphedema, Pak et al. (2021) reported better outcomes with combined LNVA and LVA than with LVA alone, but the non-randomized peripheral study cannot establish an AD indication or isolate the effect of LNVA. Figure 3 depicts the node-vein interface.

**Figure 3:**
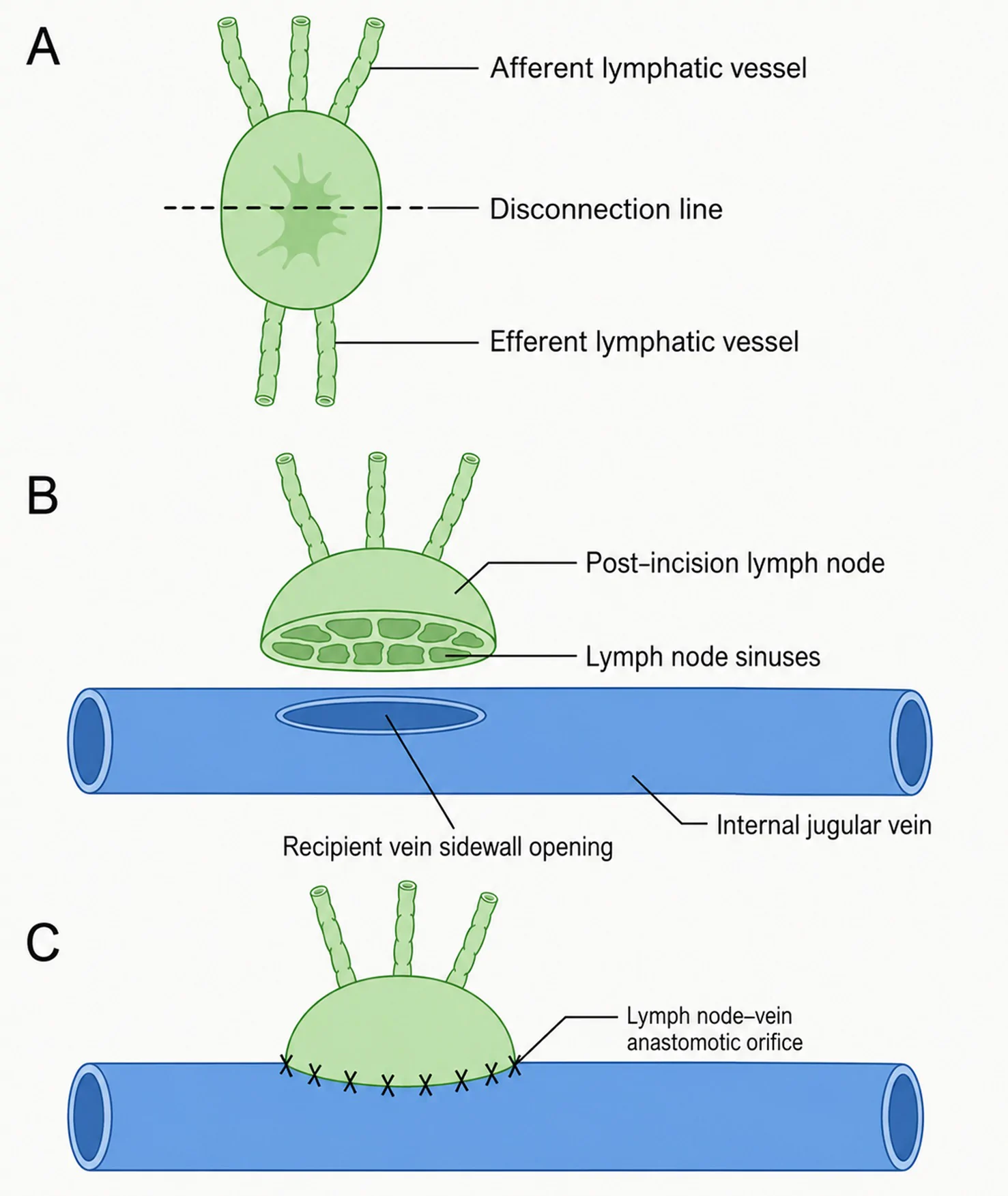
Lymph node-vein interface. **(A)** Intact lymph node with afferent and efferent lymphatics and the proposed incision line. **(B)** Incised node with exposed sinuses positioned above a venous side-wall opening. **(C)** Nodal surface sutured to the venous opening to create a sinus-to-vein interface. Function depends on a node that remains patent and sufficiently low resistance.

In AD, nodal suitability cannot be assumed. Papp et al. (1971) showed that lymph trunks and nodes have measurable, non-linear resistance *in situ* and that normal nodal resistance should not be equated with pathological obstruction. Browse et al. (1984) found that resistance rises at low flow and with venous back pressure. In an *in situ* rabbit hind-limb preparation, Nagai et al. (2008) showed that nodal flow changed with infusion pressure, downstream conditions, and acute inflammation; the model does not define human cervical pressure thresholds. LNVA leaves the nodal resistance unit immediately upstream of the anastomosis. This matters because cervical nodes may carry or process brain-related proteins (Nauen and Troncoso, 2022; Al-Diwani et al., 2025) and may show altered shape or microvascular signal in amyloid-positive AD (Kim et al., 2026). These findings challenge the assumption of a uniformly normal low-resistance nodal basin without showing that every node is obstructed or unsuitable.

Functional screening is therefore required. In 30 patients with lower-extremity lymphedema, Alshomer et al. (2024) identified ICG uptake in 22 target nodes; high-frequency ultrasound achieved 100% specificity and 100% positive predictive value for ICG-positive nodes in that cohort, compared with lower performance for lymphoscintigraphy and magnetic resonance lymphangiography. The estimates are cohort-specific and do not measure brain-derived lymph flux, transnodal resistance, or an AD surgical threshold. A visible node should not be considered functional unless target uptake, transnodal flow, venous-pressure compatibility, and durable postoperative patency can be demonstrated.

Tang et al. (2025) reported the most directly relevant AD/dementia cohort for the node-vein interface. The 28-patient exploratory study used ICG to select fluorescent, elastic nodes and connected them end-to-side to EJV branches or the EJV. Approximately 64.3% of patients had a higher Mini-Mental State Examination (MMSE) score at 1 week, and tau-related signal was detected in excised abnormal nodes. The cognitive interval is too short to separate treatment effects from perioperative state, repeated testing, expectation, or regression to the mean. Nodal tau shows arrival or retention of tau-related material, not durable brain clearance. The study supports technical and mechanistic hypothesis generation rather than efficacy or long-term patency.

The APP/PS1 model of Liu et al. (2026) adds direct experimental target engagement for a node-EJV interface: short-term cisterna-magna ICG clearance increased after side-to-side anastomosis. It does not resolve whether a human AD node is low resistance, whether net protein export increases, or whether the interface remains patent and safe over clinically relevant intervals.

### 4.3 Collecting lymphatic vessel-vein interface

Of the three interfaces, collecting lymphatic vessel-vein anastomosis is anatomically the most specific because it connects a defined lymphatic lumen directly to a defined venous lumen. Collecting vessels comprise valve-bounded pumping segments, or lymphangions, whose output depends on valves, rhythmic smooth-muscle contraction, preload, venous afterload, and mechanical signaling; sustained high pressure or reverse flow can impair valve function, contractility, and barrier integrity (Scallan et al., 2016; Angeli and Lim, 2023). Davis et al. (2025) emphasized that transport, filtering, and immune traffic are coupled across initial lymphatics, collecting vessels, nodes, and venous return, so a patent bypass may change biological handling as well as fluid resistance. Yoon et al. (2024) showed that downstream deep cervical collecting vessels have long lymphangions, semilunar valves, and smooth-muscle coverage expected of active conduits. A patent anastomosis alone does not establish restoration of the glymphatic-lymphatic axis; this interface provides the most clearly defined construct for testing directional outflow and target-level patency. Figure 4B depicts the lumen-to-lumen connection.

**Figure 4:**
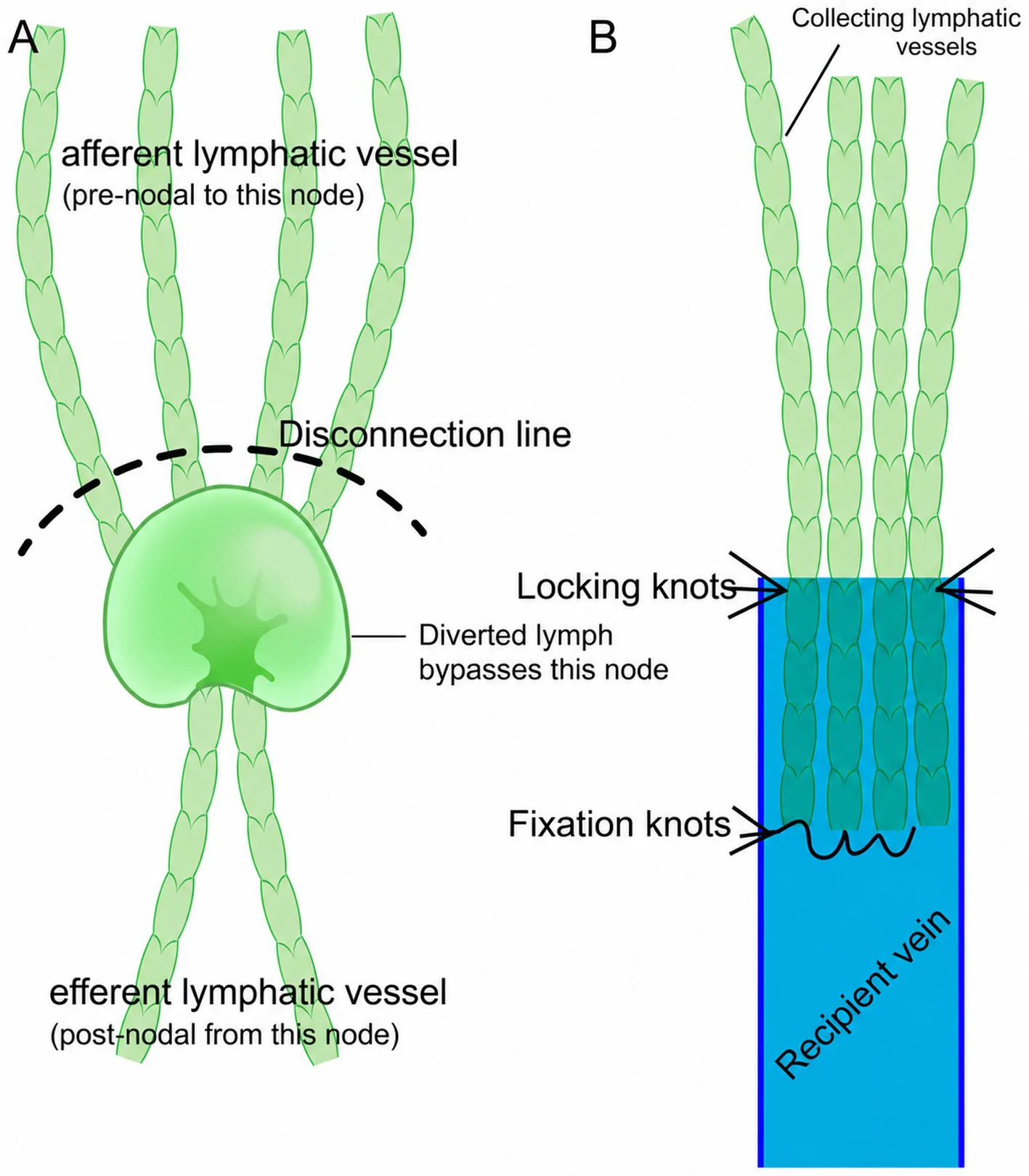
Collecting lymphatic vessel-vein interface. **(A)** Valved collecting lymphatic vessels are shown in relation to an illustrated reference node. The entering vessels are afferent/pre-nodal relative to that node; division at the indicated line and implantation of the inflow-connected ends into a vein diverts lymph without traversing the illustrated node. The vessel leaving the node is efferent/post-nodal relative to it; venous diversion from an efferent vessel would occur after lymph has traversed that node. These terms describe local anatomy, not separate operative categories: a collector connecting two nodes can be efferent from the upstream node and afferent to the downstream node. **(B)** A representative overlapping/lockup construction connects collecting lymphatic vessel lumens to a recipient vein. Locking knots secure the lymphatic ends at the venous entry, and fixation knots stabilize the overlap. The schematic defines the connected structures, not whole-network nodal bypass, preserved filtering, or immune safety; flow direction and patency require verification.

Afferent/pre-nodal and efferent/post-nodal describe the relation to a specified lymph node. Diversion of an afferent vessel before entry into that node routes the diverted lymph to a vein without traversing that node, as illustrated in Figure 4A. Conversely, diversion from an efferent vessel occurs after the lymph has traversed the specified node. Prior nodal transit does not by itself demonstrate normal filtering, preserved immune function, or immune safety. These local distinctions do not create additional operative categories: a collector connecting two nodes can be efferent from the upstream node and afferent to the downstream node. Thus, bypass of the illustrated node does not establish that the diverted lymph has never passed through other nodes. Operative reporting should identify the directly anastomosed structure, mapped source and flow direction, and relation to a named or illustrated reference node when observable; an unreported relation should not be inferred from cervical location alone. Pan et al. (2010) described valves, ampulla-like segments, trunks, and ducts in the human head and neck, but also documented complex regional convergence, so a cervical vessel may drain non-cerebral territories. Yağmurlu et al. (2020) mapped deep cervical lymphatic structures around the internal jugular chain and carotid sheath, while Murakami et al. (1994) described collateral pathways and individual variation around the internal jugular and jugulo-omohyoid chains. Nguyen et al. (2026) further identified candidate level II nodal and venous targets in fresh cadavers, but the absence of active circulation prevents inference about *in vivo* source, direction, or pressure. These studies define a search space rather than a guaranteed brain-draining target. Collateral recruitment may preserve total flow after one route is altered or divert tracer away from the intended anastomosis; source and direction require functional mapping rather than location alone.

Supermicrosurgical construction is demanding, but existing methods show that caliber mismatch can be managed. Koshima et al. (2000) established LVA in extremity lymphedema, where a functional lymphatic and a low-pressure venous recipient were essential. In 10 patients and 123 anastomoses, Zheng et al. (2024) used 44 overlapping-lockup LVAs and reported a mean construction time of approximately 5.6 min, compared with 12.1 min for conventional end-to-end LVA, with immediate ICG flow but no evidence of long-term superiority. Wang C. et al. (2024) reported a related overlapping technique in 17 patients and approximately 194 anastomoses, again demonstrating immediate technical feasibility rather than durable patency. Operative time and intraoperative fluorescence are engineering endpoints, not evidence of cerebral clearance or clinical benefit.

Chen et al. (2026) described a dynamic neck LVA in which a lower-limb vein graft traversed the sternocleidomastoid muscle, was connected proximally to the external jugular vein, and received three collecting lymphatic vessels distally. This is best treated as a collecting-vessel-interposed vein-graft-vein construct rather than a standard direct LVA. The proposed movement- or Venturi-assisted mechanism remains unverified and requires direct pressure-flow, directional-flow, and reflux testing.

Ma et al. (2025) reported a 41-patient, three-month retrospective clinical and biomarker study that is mapped in Table 2 to a lymphatic vessel-vein interface. Approximately 50% of patients met the study’s Clinical Dementia Rating-Sum of Boxes (CDR-SB) response definition, more than two-thirds had at least one Aβ42/40 or tau phosphorylated at threonine 181 (p-tau181) measure reported in a favorable direction, and two mild adverse events occurred. Shorter disease duration in Ma et al. may also identify patients with a better baseline prognosis. Fu et al. (2026) described anastomosis of branches of deep cervical lymphatic vessels to openings in the EJV or its branches. Because the structures directly connected to the venous lumen were lymphatic vessels rather than a lymph node or lymphatic tissue flap, that study is also classified as a collecting lymphatic vessel-vein interface. Partial regional lymph-node resection was also reported; this co-intervention may affect nodal handling but does not change the identity of the described vessel-to-vein anastomoses. The local relation of each anastomosed collector to individual nodes was not sufficiently detailed to infer which nodal transit was retained or bypassed. Without randomized treatment comparisons, durable target-level patency, and source attribution, these reports cannot establish efficacy or disease modification.

Peripheral LVA evidence informs technique, not AD efficacy. Verhey et al. (2022) reviewed 74 studies involving approximately 6,260 patients and found reported objective improvement ranging from 23.3 to 100%, reflecting wide variation in disease stage, procedure, compression therapy, outcome definition, and follow-up. Meta-analyses report signals in limb volume, cellulitis, and quality of life but remain dominated by observational before-after studies (Meuli et al., 2023; Zurfluh et al., 2025). The latter review included 74 studies and 4,692 patients, with pooled improvements extending beyond volume reduction to quality of life and fewer cellulitis episodes, but no evidence of a cerebral mechanism. The collecting vessel-vein interface merits standardized testing because it is anatomically defined and directly measurable; peripheral success provides technical precedent, not evidence of brain-protein clearance or cognitive benefit. Figure 5 provides a mechanistic comparison of the three target-defined interfaces and their validation requirements.

**Figure 5:**
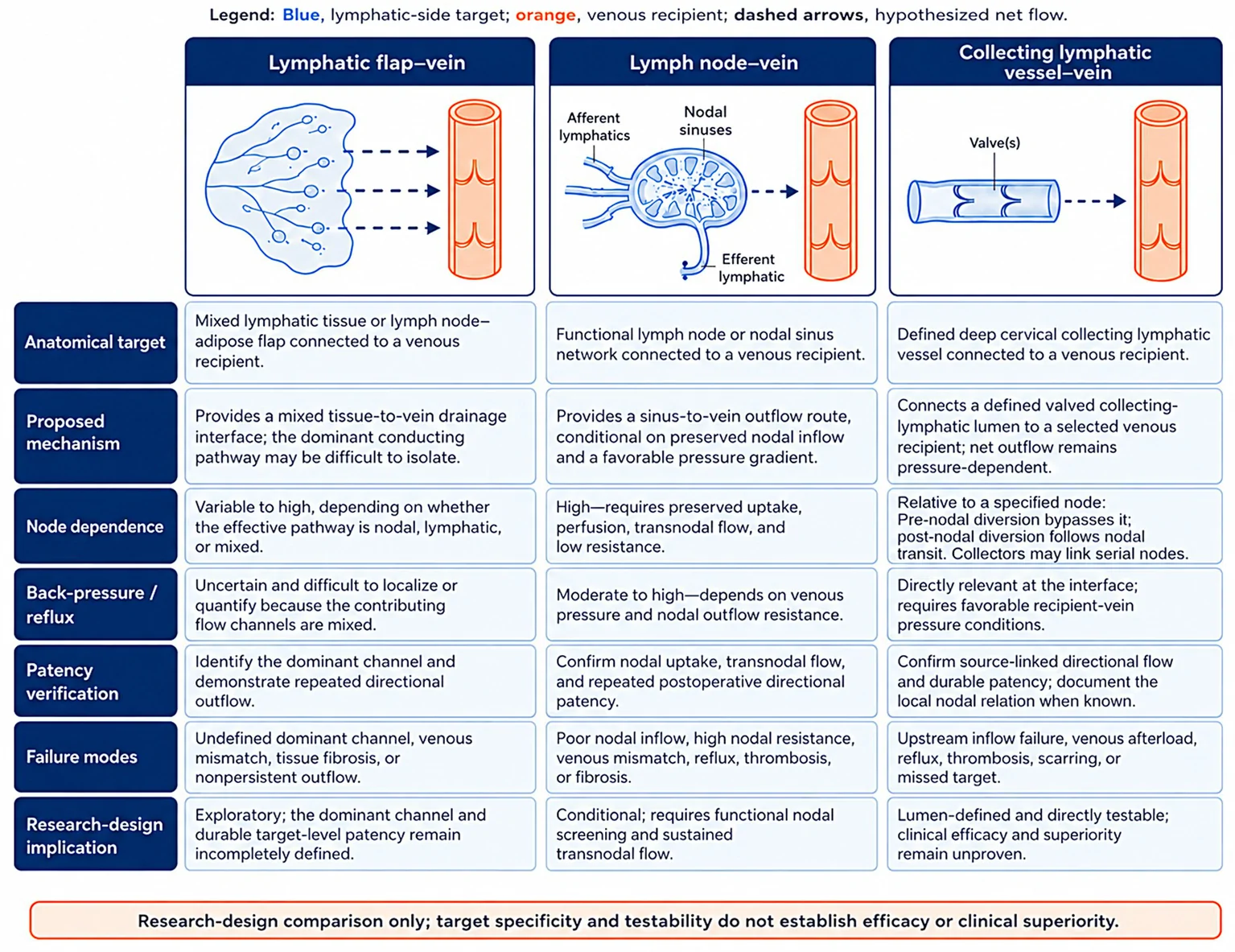
Mechanistic comparison of the three target-defined interfaces. Blue denotes the lymphatic-side target, orange denotes the venous recipient, and dashed arrows indicate hypothesized net flow. The comparison covers anatomical target, proposed mechanism, dependence on lymph node function, venous back-pressure or reflux, patency verification, failure modes, and research-design implications. For the collecting lymphatic vessel-vein interface, nodal dependence is network- and node-relative: diversion before a specified node bypasses that node for the diverted lymph, whereas diversion after it follows prior transit through that node. A collector connecting serial nodes can have both relations; these are anatomical descriptions, not additional interface categories. Prior nodal transit does not establish normal filtering or immune safety. The schematics are conceptual and not anatomically to scale. Target specificity and testability guide study design and do not establish efficacy or clinical superiority.

## 5 Human evidence and translational boundaries

### 5.1 Early clinical studies and registered trials

Human studies currently occupy four distinct evidentiary levels: operative feasibility, local or inferred patency, biological or imaging response, and patient-level outcome. Most AD reports are case studies, single-arm cohorts, retrospective analyses, or pilots, and dcLVA/LVA does not denote a standardized operation. The evidence supports feasibility and hypothesis generation; durable target-level patency linked to blinded patient outcomes remains largely untested.

Xie et al. (2024) outlined a supermicrosurgical research agenda but provided no controlled efficacy data. Li X. et al. (2024) described one patient with short-term changes in MMSE, CDR-SB, tau-PET, and fluorodeoxyglucose PET (FDG-PET) 5 weeks after cervical shunting. The case established operability and generated a hypothesis, but it lacked a counterfactual and was vulnerable to expectation, intensified postoperative care, sleep and medication changes, repeated testing, regression to the mean, and natural within-person variability. The operative interface was also too incompletely described for target-specific causal inference.

Chen J. Y. et al. (2025) reported a prospective 26-patient single-arm cohort with one-month follow-up. Approximately 60% of caregivers reported symptomatic improvement, the median MMSE increased from 3 to 5, CSF biomarker changes were non-significant, and two patients developed transient difficulty raising an arm that subsequently improved. The procedure is mapped here to a lymphatic flap-vein configuration based on the operative description and figures. The study documents technical feasibility and short-term tolerability, but the two-point MMSE change is within the range of measurement error reported in moderate-to-severe dementia, and the unblinded caregiver and assessor context prevents a treatment-effect estimate. Jin L. et al. (2025) further described the related lymph node-adipose flap/EJV bypass as a technical and monitoring framework rather than efficacy evidence.

Tang et al. (2025) reported a short-term 28-patient dementia cohort mapped in Table 2 to a lymph node-vein interface. The operation used ICG-guided selection of fluorescent, elastic nodes and end-to-side anastomosis to EJV branches or the EJV. A one-week MMSE increase was reported in approximately 64.3% of patients, and tau-related signal was detected in excised abnormal nodes. The first finding is too early and uncontrolled to distinguish treatment from perioperative and measurement effects; the second shows that cervical nodes can carry or retain tau-related material but does not establish that the operation durably reduces brain tau. Al-Diwani et al. (2025) similarly showed biomarker enrichment in node aspirates, supporting nodal accessibility and processing rather than surgical efficacy.

Two larger cohorts extend the feasibility evidence without resolving causal attribution. Ma et al. (2025) reported a 41-patient retrospective study in mild-to-moderate AD with three-month clinical and biomarker signals, including an exploratory 50% response definition and favorable movement in at least one Aβ42/40 or p-tau181 measure in more than two-thirds of patients; the design, multiple endpoints, and missing target-level patency data preclude efficacy inference. Fu et al. (2026) followed 139 patients with severe AD for 6 months and reported modest cognitive, functional, and fluid-biomarker changes. No deaths occurred, but perioperative delirium and sleep disturbance were reported and one patient required reoperation for bleeding or hematoma. The single-arm design, floor and practice effects, uncertain source of peripheral biomarker changes, and lack of durable patency assessment prevent attribution to a durable, source-defined collecting lymphatic vessel-vein reconstruction. The relation of the anastomosed collectors to individual cervical nodes was not sufficiently reported for a node-relative anatomical description. Fu et al. reported assessor blinding and a descriptive comparison with an external lecanemab cohort; those features do not supply a randomized or concurrent within-study control.

Chen et al. (2026) reported five patients treated with a dynamic neck vein-graft construct. All grafts were described as patent and no postoperative complications were recorded; mean MMSE and Montreal Cognitive Assessment (MoCA) changes were +1.6 and +1.4, the Neuropsychiatric Inventory (NPI) decreased by 5.8, the Clinical Dementia Rating (CDR) was unchanged, and PET changes varied by patient. The very small uncontrolled sample, multiple endpoints, short follow-up, and measurement-sensitive cognitive changes preclude estimates of efficacy or uncommon harm. Observed short-term patency also does not validate durable directional outflow or the proposed dynamic mechanism.

Jin L. et al. (2026) retrospectively compared six-month lecanemab and dcLVA cohorts using AV-45 PET. Cognitive decline was reported in 23.5 and 81.8%, respectively; change in centiloid differed between groups, whereas change in standardized uptake value ratio (SUVR) did not. This comparative signal argues against assuming equivalence or superiority of dcLVA, but the non-randomized design, unclear operative interface and durable patency, possible treatment-selection confounding, and internally derived imaging threshold prevent a causal comparative-effect estimate.

Hu et al. (2026) added an exploratory single-arm biomarker study of 30 patients followed for 180 days. Plasma Aβ42 increased and correlated with the Clinician’s Interview-Based Impression of Change plus caregiver input (CIBIC-plus), while the combination of Aβ42 and Aβ42/40 yielded an AUC of 0.737 for the study’s clinical classification at 180 days. This is moderate, internally derived discrimination rather than a validated decision threshold. The absence of an untreated comparator, target-level patency, external validation, and complete interface disclosure means the study cannot identify whether the change reflects brain export, platelet or peripheral production, renal handling, inflammation, or analytic variability. Its value is to justify serial sampling and pre-specified mechanistic hypotheses, not to establish enhanced cerebral clearance or clinical efficacy.

Baseline phenotype has also been explored as a possible effect modifier. In a retrospective pilot with only 10 analyzed patients, Wu X. et al. (2025) classified 4 as short-term responders and proposed PVS-volume cut points of approximately 5,150 mm^3^ for total PVS and 3,630 mm^3^ for white-matter PVS, with within-sample AUCs of 1.00 and 0.88. The thresholds and performance were derived in the same very small dataset, the response definition relied on a two-point cognitive change at 1 month, and PVS burden varies with age, hypertension, small-vessel disease, and segmentation methods. The study supports the hypothesis that baseline clearance phenotype may modify apparent response, but does not provide a clinical action threshold, independent evidence of efficacy, or durable patency data.

Table 2 places these reports and registrations along the continuum from feasibility to validation.

These studies remain in the early-development phase of surgical innovation. The Idea, Development, Exploration, Assessment, and Long-term study (IDEAL) framework calls for prospective registration of consecutive cases and transparent reporting of technical changes, operator learning, patient selection, failures, and harms before comparative efficacy claims are made (McCulloch et al., 2013). The Strengthening the Reporting of Cohort, Cross-sectional and Case–control Studies in Surgery (STROCSS) 2024 guideline similarly requires clear reporting of center and time frame, enrollment, intervention details, surgeon experience, confounding, missing data, loss to follow-up, and complications in observational surgical studies (Rashid et al., 2024). Randomized evaluation should follow the Consolidated Standards of Reporting Trials extension for nonpharmacologic treatments (CONSORT-NPT) requirements for procedural standardization, operator expertise, center effects, co-interventions, adherence, feasible blinding, and complete harms reporting (Boutron et al., 2017). A prospective randomized open blinded-endpoint (PROBE) design permits open surgery with central randomization and blinded adjudication, but cannot remove expectation bias from caregiver- or patient-reported outcomes (Hansson et al., 1992).

The registered Deep Cervical Lymphatic Venous Anastomosis in the Treatment of Alzheimer’s Disease (CLEAN-AD) studies move the field toward standardized evaluation but have not yet reported results. CLEAN-AD is planned as a multicenter randomized parallel study of approximately 376 patients, with 12-month cognitive, functional, safety, imaging, and biomarker outcomes; the open-label operation and blinded-endpoint design address some, but not all, expectation and center effects (ClinicalTrials.gov, 2026a). The CLEAN-AD Registry plans approximately 814 patients across about 40 centers with follow-up to 24 months and a broad set of clinical, imaging, fluid-biomarker, caregiver, and safety measures (ClinicalTrials.gov, 2026b). The registry can estimate heterogeneity, rare adverse events, learning curves, and external validity, but without a non-surgical comparator it cannot convert before-after change into a causal treatment effect. Both records remain research plans, and neither currently provides enough target-level operative detail or patency results to validate one of the three interfaces.

### 5.2 Biomarker interpretability and surrogate-endpoint boundaries

Changes over days or weeks must be interpreted against the measurement properties of cognitive scales. In 130 community participants retested after 2–4 months, Feeney et al. (2016) estimated that an individual change of at least 3 MMSE points or 4 MoCA points was needed to exceed measurement error with reasonable confidence. In 88 nursing-home residents with dementia and a mean MMSE of approximately 14, Hörnsten et al. (2021) found a minimum detectable MMSE change of 4 points over 1–6 days, with individual differences of up to 6 points. Galasko et al. (1993) demonstrated practice effects after repeated MMSE exposure in AD, particularly during early retesting. These values are not universal thresholds for clinically important change, but one- to two-point shifts over short intervals in uncontrolled studies remain measurement-sensitive and should not be interpreted as neural recovery or disease modification.

Postoperative biomarker changes indicate biological response, not necessarily disease modification. Plasma tau phosphorylated at threonine 217 (p-tau217) has high diagnostic value for biological AD: Therriault et al. (2023) reported an AUC of approximately 0.91 for plasma p-tau217 versus 0.94 for CSF p-tau217 in identifying amyloid PET positivity, and Ashton et al. (2024) confirmed strong performance of a commercial assay across clinical and research cohorts. These studies support pathology confirmation and trial-entry stratification; they do not show that a short-term postoperative change reflects enhanced clearance. Yang et al. (2023) found that glial fibrillary acidic protein (GFAP) combined with p-tau181 identified amyloid positivity with AUCs of approximately 0.86 overall and 0.93 in cognitively impaired participants. GFAP primarily reflects astroglial reactivity and may also change with surgery, inflammation, renal function, or vascular injury. Each marker should therefore be tied to the biological process it represents and interpreted alongside anatomy, directional patency, imaging, and blinded outcomes.

Li Y. et al. (2026) analyzed preoperative CSF from 90 patients and reported 16 proteins that differed between study-defined responders and non-responders. A model combining neuronal pentraxin-2 (NPTX2) and immunoglobulin superfamily member 10 (IGSF10) yielded an AUC of 0.854; adding p-tau181 and Aβ42 increased the AUC to 0.880, with fivefold cross-validation AUC 0.859 ± 0.074. These are internally derived prognostic candidates, not proof of a drainage mechanism or a validated surgical selection threshold, because the response definition, untreated comparison, external validation, and target-specific patency remain unresolved.

DTI-ALPS and related imaging measures are also indirect. Huang et al. (2024) found that lower DTI-ALPS predicted greater amyloid deposition, neurodegeneration, and subsequent clinical progression, while Li Y. et al. (2024) replicated lower DTI-ALPS in subjective cognitive decline across two cohorts. These associations support risk stratification and longitudinal hypothesis testing. DTI-ALPS, however, is influenced by white-matter fiber geometry, small-vessel disease, atrophy, motion, and scanner protocol and does not measure net CSF or cervical lymphatic flow. A postoperative increase is interpretable only if it accompanies target-level directional patency, downstream cervical evidence, and concordant clinical or pathological change.

### 5.3 Safety and regulatory boundaries

The regulatory context sharply limits clinical interpretation. A 2025 notice from the General Office of the National Health Commission described deep cervical lymphatic vessel/node-vein anastomosis for AD as an early exploratory procedure with unclear indications and contraindications and insufficient high-quality evidence of safety or efficacy. It prohibited clinical treatment use and required adequate relevant preclinical evidence and ethics-committee review before clinical research (National Health Commission of the People’s Republic of China, 2025). The notice defines an evidence and safety boundary; it does not resolve every clearance hypothesis. Jiang et al. (2026) later described a 67-year-old patient with approximately 2 mL of intraventricular hemorrhage 23 days after bilateral dcLVA, suspected Epstein–Barr virus reactivation, and a subsequent seizure disorder accompanied by a positive tissue-based neural antibody assay. Operative details were incomplete and temporal association does not establish causality, but the case identifies delayed harms that small single-arm series may miss.

Prospective protocols need safety surveillance beyond immediate surgical complications. Prespecified local and neurological outcomes should include hemorrhage, thrombosis, reflux, occlusion, fibrosis, reintervention, loss of net outflow, delayed seizures, and immune-mediated neurological events. Flow and reflux assessments should use standardized postural and venous-pressure conditions. Consent, caregiver expectation, monitoring capacity, and delayed follow-up are particularly important in cognitively impaired patients.

Systemic consequences are also uncertain. Direct node- or tissue-to-vein constructs, and collecting-vessel diversion performed before entry into a specified node, may alter antigen capture, local immune-cell trafficking, or the timing and concentration with which CNS-derived proteins, inflammatory mediators, cells, or pathogens enter the venous circulation. For a collecting-vessel interface, diversion before a specified node bypasses that node for the diverted lymph, whereas diversion from its efferent vessel follows transit through that node. A collector connecting serial nodes can have both relations, so the interface label alone cannot establish how much nodal processing precedes venous entry. Prior transit must not be equated with normal filtering or proven immune safety. These are mechanistic possibilities rather than established adverse effects, and their direction and clinical magnitude are unknown. Baseline cerebral amyloid angiopathy risk, vascular fragility, antithrombotic exposure, immune status, active infection or recent viral reactivation, systemic inflammatory or autoimmune disease, clinically significant immunosuppression, active malignancy, and capacity for prolonged follow-up should be documented prospectively.

Long-term immunological surveillance should be interface-specific, anatomically documented, and longitudinal. Direct node- or tissue-to-vein constructs may affect nodal antigen presentation and immune-cell trafficking more directly. For collecting lymphatic vessel-vein anastomosis, surveillance should account for the documented local nodal relation and any concomitant nodal resection rather than presume uniform immune consequences. Protocols should therefore record the directly anastomosed structure, mapped source and flow direction, and local relation to adjacent nodes when observable, and should obtain baseline data with repeat clinically adjudicated assessment at 3, 6, and 12 months and, where feasible, annually thereafter. Minimum domains should include serious or recurrent infection, viral reactivation, new autoimmune or immune-mediated neurological events, persistent inflammatory or hematologic abnormalities, thrombotic or vascular events, and organ dysfunction. Serial blood counts and selected inflammatory or immune markers may support interpretation, but isolated laboratory shifts should not be labeled benefit or toxicity without clinical correlation. Controlled comparators and independent event adjudication are important because aging, AD, comorbidity, and perioperative care can produce the same outcomes.

## 6 Mechanistic validation framework for aging neuroscience

### 6.1 Target suitability and patient stratification

Technical access to a lymph node is not the main uncertainty; nodal function is. A deep cervical node in AD may not remain perfused, low resistance, non-inflamed, and connected to upstream flow. Aβ and other neurodegenerative biomarkers are enriched in cervical nodes (Nauen and Troncoso, 2022; Al-Diwani et al., 2025), which establishes biological contact with the pathway but not suitability for bypass.

AD-specific imaging reinforces the need for individual assessment. Kim et al. (2026) compared 25 amyloid PET-positive patients with AD with 25 controls and assessed 482 deep cervical nodes. AD-associated nodes were less often regularly oval, more often had poorly defined internal architecture, and showed reduced microvascular signal; an exploratory composite model produced an AUC of 0.81, with 64% sensitivity and 84% specificity. Multiple nodes from the same participant are not independent observations, and the study does not establish obstruction, fibrosis, or LNVA failure. It does show that location alone is an inadequate basis for target selection.

Pressure-flow data reinforce this concern. Papp et al. (1971) demonstrated variable resistance in lymph trunks and nodes *in situ*. Browse et al. (1984) measured a decline in mean nodal resistance from approximately 180 mmHg/mL/min at flows below 0.1 mL/min to approximately 68 above 1.0 mL/min, with an increase of about 8.6 resistance units for each 10-mmHg rise in venous pressure. Nagai et al. (2008) showed that perfusion pressure, outflow pressure, and acute inflammation reshape nodal flow. These animal values are not clinical thresholds, but they show that a node can become a dynamic bottleneck in the low-flow, pressure-sensitive range relevant to cervical lymphatic outflow.

Peripheral LNVA offers a screening strategy, not an AD indication. Bailey et al. (2024) emphasized preoperative mapping, node quality, recipient-vein selection, technical refinement, and operator expertise. In 160 patients with lower-extremity lymphedema, Pak et al. (2021) reported greater improvement with LNVA plus LVA than with LVA alone, but the retrospective non-randomized design and peripheral disease context prevent isolation of the nodal effect. In a 30-patient peripheral cohort, Alshomer et al. (2024) found 22 ICG-positive target nodes and reported 100% specificity and positive predictive value for high-frequency ultrasound in identifying them. The findings may reduce unproductive exploration, but do not measure brain-derived flow, transnodal resistance, or an AD surgical target.

The evidence does not show that LNVA must fail in AD. It shows that the target node cannot be assumed to be normal, low resistance, or durable. LNVA is therefore a conditional research path that requires functional screening before or during surgery and postoperative evidence of sustained patency and net outflow.

AD diagnosis alone is insufficient for trial entry. A research-only selection sequence should separate four questions. First, is AD biologically confirmed by amyloid or tau PET, CSF, or a validated plasma marker such as p-tau217? Second, is there a reproducible clearance-axis abnormality, assessed with complementary central measures such as DTI-ALPS or PVS burden together with sleep, vascular, and small-vessel-disease characterization rather than any single surrogate? The diagnostic performance reported for p-tau217 supports entry classification but does not validate treatment response, and DTI-ALPS remains sensitive to white-matter anatomy, atrophy, motion, vascular disease, and scanner parameters. The CSF NPTX2/IGSF10 panel reported by Li Y. et al. (2026) is likewise a candidate predictor derived within one cohort, not a validated eligibility threshold.

Third, can a downstream cervical target be mapped and shown to carry directional flow by magnetic resonance lymphatic imaging, ultrasound, ICG or related intraoperative mapping where appropriate, with assessment of node morphology and perfusion or a defined collecting-vessel source? Fourth, is the proposed recipient vein compatible under standardized supine and dynamic postural or respiratory conditions, without relevant reflux or unstable outflow? A central clearance abnormality without demonstrable cervical dysfunction does not justify a cervical operation; conversely, a cervical abnormality without evidence of coupling to central clearance remains a target-identification signal rather than proof of an AD mechanism. No imaging, biomarker, flow, or pressure measure currently provides a validated AD-specific surgical threshold.

For prospective research, the four questions can be converted into a proposed minimum trial-entry gate. Enrollment should require all four domains: biomarker-confirmed AD; a reproducible clearance-axis phenotype supported by complementary modalities or repeat testing under standardized conditions; an interface-specific cervical target with a defined upstream source and demonstrable directional flow; and a recipient vein with acceptable pressure and reflux behavior together with capacity for blinded longitudinal follow-up. Target evidence should match the proposed interface: flap-vein studies should identify the dominant outflow channel; LNVA studies should document nodal uptake, perfusion, and transnodal flow; and collecting-vessel studies should define a source-linked valved lumen and report its local relation to adjacent nodes when technically observable. Absence or unresolved discordance in any domain should defer enrollment rather than be offset by a favorable cognitive score or an isolated biomarker. Protocols should prespecify thresholds, adjudication rules, handling of missing data, and go/no-go criteria; none is currently validated for routine clinical care.

Candidate exclusion or stratification variables include major cervical distortion from surgery or radiotherapy, high thrombosis risk, unstable venous outflow, active infection or recent viral reactivation, uncontrolled systemic inflammatory or autoimmune disease, clinically significant immunosuppression, active malignancy, inability to complete serial imaging and long-term safety follow-up, absence of biological AD confirmation, and cognitive or behavioral states that undermine blinded outcome assessment. These are research eligibility considerations, not recommendations for clinical use.

Table 3 separates direct evidence on cervical nodes from cross-level inference.

**Table 3:** Evidence relevant to cervical lymph nodes as operative targets.

Evidence layer	Representative sources	Direct support	Does not establish by itself	Interpretive relevance
Human cervical nodal burden	Nauen and Troncoso (2022); Al-Diwani et al. (2025)	Aβ-positive cells were >40-fold enriched in cervical versus inguinal opportunity specimens; living-node aspirates can enrich multiple neurodegenerative markers relative to plasma.	Effective clearance flux, inevitable obstruction, fibrosis, source specificity, or a validated surgical surrogate.	Establishes a human biological compartment and weakens the default assumption of an unloaded, uniformly low-resistance node.
AD-specific nodal phenotype	Kim et al. (2026)	In 25 amyloid-positive AD participants and 25 controls (482 nodes), morphology and microvascular signal differed; exploratory composite AUC 0.81.	Causal obstruction, diagnostic validity, LNVA failure, or a surgical action threshold.	Supports independent target-node assessment and prospective external validation.
Pressure-flow evidence	Papp et al. (1971); Browse et al. (1984); Nagai et al. (2008)	Nodes are dynamic resistance units; resistance is greatest at low flow, rises with venous back pressure, and changes with inflammation.	Human AD cervical pressure thresholds or modern LNVA patency rates.	Identifies a potentially unstable resistance segment upstream of the anastomosis.
Peripheral LNVA analogy	Pak et al. (2021); Alshomer et al. (2024); Bailey et al. (2024)	Peripheral LNVA depends on functional-node imaging, preserved lymphatic input, recipient-vein selection, technical refinement, and operator expertise.	Brain-derived lymph flux, AD efficacy, or clinical priority.	Defines a screening and reporting logic while preventing direct efficacy extrapolation.
AD mouse target-engagement model	Liu et al. (2026)	Side-to-side deep cervical node-EJV anastomosis increased short-term clearance of cisterna-magna ICG signal in 10-month-old male APP/PS1 mice.	Human efficacy, protein flux, long-term patency, immune safety, or cognitive benefit.	Provides an experimental platform for mechanism testing while requiring human pressure-flow and durability validation.
Fresh-cadaver anatomical mapping	Nguyen et al. (2026)	Dural/parietal ICG, histology, and simulated robotic LNVA identified a posterior route toward level II nodes and adjacent veins.	*In vivo* directional flow, active pumping, pressure gradients, a rate-limiting bottleneck, or patient-selection threshold.	Expands the anatomical search space but must be verified with living human functional measurements.

Direct observations are separated from cross-level inference. AD, Alzheimer’s disease; Aβ, amyloid beta; APP/PS1, amyloid precursor protein/presenilin-1; AUC, area under the receiver operating characteristic curve; EJV, external jugular vein; ICG, indocyanine green; LNVA, lymph node-vein anastomosis.

### 6.2 Durable target engagement and longitudinal assessment

Medium-term patency is necessary before an LNVA-associated clinical, imaging, or biomarker change can be interpreted as sustained decompression or clearance. Nodal and lymphatic-venous interfaces remodel over weeks to months. Three months is therefore a reasonable minimum assessment point, with six and 12 months needed to test sustained flow and safety.

Calnan et al. (1967) studied canine popliteal lymph node-to-vein anastomosis. Patency and function were present at approximately 2 weeks but were lost by two to 3 months under the experimental conditions, with endothelial growth and intimal-like fibrosis. The model does not show that modern cervical LNVA inevitably fails; it shows that early flow cannot replace medium-term patency testing.

Patency is possible under favorable conditions, but it remains an empirical outcome. Nielubowicz and Olszewski (1968) demonstrated sustained flow in selected experiments. In 57 canine microsurgical lymphovenous anastomoses, Gloviczki et al. (1986) found 8 of 10 patent at 24 h, 8 of 19 at 2–6 weeks, 4 of 6 at 3 months, and 2 of 6 at 8 months; venous thrombosis was a major failure mechanism and antiplatelet treatment did not clearly prevent closure. Yamada (1969) also reported high early patency followed by lower six-month patency in historical lymphedema models. The models are technically dated and non-cerebral, but they show that constructing an anastomosis does not ensure durability.

Human peripheral lymphedema studies also support repeated, objective patency assessment. In 18 patients and 67 upper-extremity anastomoses, Suzuki et al. (2019) found clear six-month patency in 14 of 44 side-to-end and 8 of 23 end-to-end anastomoses, approximately 32 and 35%. These rates do not estimate cervical patency but refute the assumption that intraoperative ICG passage predicts durable function. Maegawa et al. (2012) reported 107 patients who underwent 472 lymphaticovenous side-to-end anastomoses; among 57 patients with 223 evaluable anastomoses, cumulative ICG-assessed patency was approximately 75% at 12 months and 36% at 24 months. Limb-volume change did not differ significantly between those with patent and without clearly patent anastomoses, and deep-thigh targets were difficult to assess by near-infrared imaging. Subjective improvement cannot replace repeated target-level flow measurement, and local patency must still be linked to disease-relevant biological and clinical effects.

Nodal biology adds uncertainty beyond simple patency. Lymph nodes contain subcapsular, cortical, and medullary sinus networks that slow, filter, and present antigens before a smaller number of efferent vessels carry lymph onward (Bujoreanu and Gupta, 2026). Opening or bypassing a node may therefore alter immune processing as well as pressure. Ulvmar and Makinen (2016) showed that lymphatic endothelium differs across initial lymphatics, collecting vessels, valves, and nodal sinuses because developmental origin and local signals shape permeability, contraction, and immune traffic. A nodal sinus surface is not biologically or mechanically equivalent to a collecting-vessel lumen.

The venous recipient is also variable. Valdueza et al. (2000) showed that internal jugular flow predominates when supine but shifts toward vertebral venous pathways after standing. Gisolf et al. (2004) demonstrated that posture and central venous pressure redistribute cerebral venous outflow. Three-dimensional low-field MRI showed progressive and asymmetric internal-jugular collapse with increasing inclination (Van Zandwijk et al., 2022). In 19 healthy participants, Laganà et al. (2017) documented cardiac, respiratory, and postural influences on internal-jugular and vertebral venous return. These studies did not measure lymph, but they show that a recipient vein is not a constant low-pressure reservoir. Patency assessment should therefore use standardized and, where feasible, dynamic postural and respiratory conditions.

Flow should be reassessed at three, six, and 12 months. Registry protocols should report these time points explicitly when they are included (ClinicalTrials.gov, 2026b). The relevant endpoint is not an intraoperative visible connection, but directional flow that remains objectively demonstrable months later and coincides with fluid-biomarker and imaging changes.

### 6.3 Comparative research priority and technical accessibility

Using sustained, low-resistance, directional outflow at three to 6 months as the mechanistic endpoint places collecting lymphatic vessel-vein anastomosis first for standardization because its interface is most specific and directly testable. This is a methodological ordering, not a treatment recommendation.

Human imaging shows that the downstream route can be reached and measured, but not that surgery improves total clearance. Eide et al. (2018) used serial MRI after intrathecal gadobutrol in clinically selected patients and observed delayed cervical-node enhancement associated with intracranial tracer kinetics. The invasive tracer, selected population, signal-intensity method, and inability to calculate absolute protein flux limit interpretation. Albayram et al. (2022) provided a non-invasive structural imaging approach without directly validating flow direction or quantity. Jacob et al. (2022) demonstrated conserved skull-base, cranial-nerve, nasopharyngeal, and cervical drainage circuits across mice and humans while emphasizing a multi-route network rather than a single duct. The studies establish anatomical accessibility and measurement hypotheses, not a guarantee that one bypass increases total net outflow.

Collecting vessel-vein anastomosis is the most specific interface because its lymphatic-side object is a valved, contractile conduit. Ma et al. (2025) and Fu et al. (2026) are mapped to this category because the reported anastomotic object is a lymphatic vessel, although neither report establishes durable source-defined flow. Angeli and Lim (2023) described how shear, stretch, matrix stiffness, and transmural pressure regulate lymphatic junctions, valves, pumping, permeability, and immune-cell traffic; sustained high pressure or reverse flow can convert a patent pathway into a dysfunctional one. Yoon et al. (2024) showed that downstream medial deep cervical vessels have the structure of active conduits and carried substantially more tracer than a lateral route in mice, whereas aging preferentially damaged the upstream nasopharyngeal plexus. An anatomically ideal distal anastomosis may therefore fail to correct an upstream input defect.

LNVA remains conditional. Nodal resistance increases with low flow and venous pressure (Browse et al., 1984) and changes with inflammation (Nagai et al., 2008). Human studies show that cervical nodes can contain Aβ, p-tau, and glial biomarkers and may have altered structure or vascularity in AD (Kim et al., 2026; Nauen and Troncoso, 2022; Al-Diwani et al., 2025). These findings do not rule out LNVA, but they require functional screening and durable patency assessment.

Lymphatic flap-vein anastomosis remains exploratory. Its practical advantage is access when individual collecting vessels cannot be identified or reproducibly anastomosed. Chen J. Y. et al. (2025) and Wu X. et al. (2025) are mapped to this category in Table 2, while Jin L. et al. (2025) provide a related bypass and monitoring concept. These reports inform technical development, but accessibility does not establish physiological priority or durable target-specific patency.

Mechanistic priority and technical accessibility may produce different orderings. Collecting-vessel reconstruction remains the most direct validation construct, LNVA is conditional on nodal screening, and flap-vein reconstruction is exploratory; technical accessibility may reverse this order. All should remain confined to appropriately approved research, with AD relevance judged by durable directional outflow linked to blinded biological and clinical endpoints rather than operative convenience (National Health Commission of the People’s Republic of China, 2025).

### 6.4 Staged validation roadmap

Before efficacy trials, the field needs consistent terminology, patient selection, patency endpoints, biomarker interpretation, and follow-up windows. The IDEAL framework requires prospective recording of consecutive cases, technical changes, operator learning, failures, and harms while the procedure is still being defined (McCulloch et al., 2013). This is particularly important when the target or operative configuration may change during development.

At the conceptual level, the pathway begins with age-related clearance dysfunction; operational validation begins with target identification. Trial entry should satisfy the four-domain research gate defined in Section 6.1. Stage 0 should establish reproducible identification of the proposed lymphatic or nodal target using magnetic resonance lymphatic imaging, ICG patterns, ultrasound, or related mapping methods. Stage 1 should test operative reproducibility, directional target-level flow, thrombosis, reflux, and perioperative safety without treating cognitive change as the primary efficacy endpoint. Stage 2 should verify durable patency at 3, 6, and 12 months and examine concordance between imaging and biomarker findings, together with delayed neurological, infectious, and immune surveillance. Stage 3 should use controlled longitudinal designs, preferably randomized or PROBE-style, with blinded patient-level outcomes, including CDR-SB, activities of daily living (ADL), NPI, caregiver burden, quality of life, and long-term safety. Progression should occur only when prespecified go/no-go criteria are met; clinical utility requires clinically meaningful, reproducible benefit and an acceptable risk–benefit balance.

Figure 6 summarizes this conditional pathway from age-related clearance dysfunction through target definition, trial-entry stratification, target engagement, controlled clinical validation, and a clinical utility gate.

**Figure 6:**
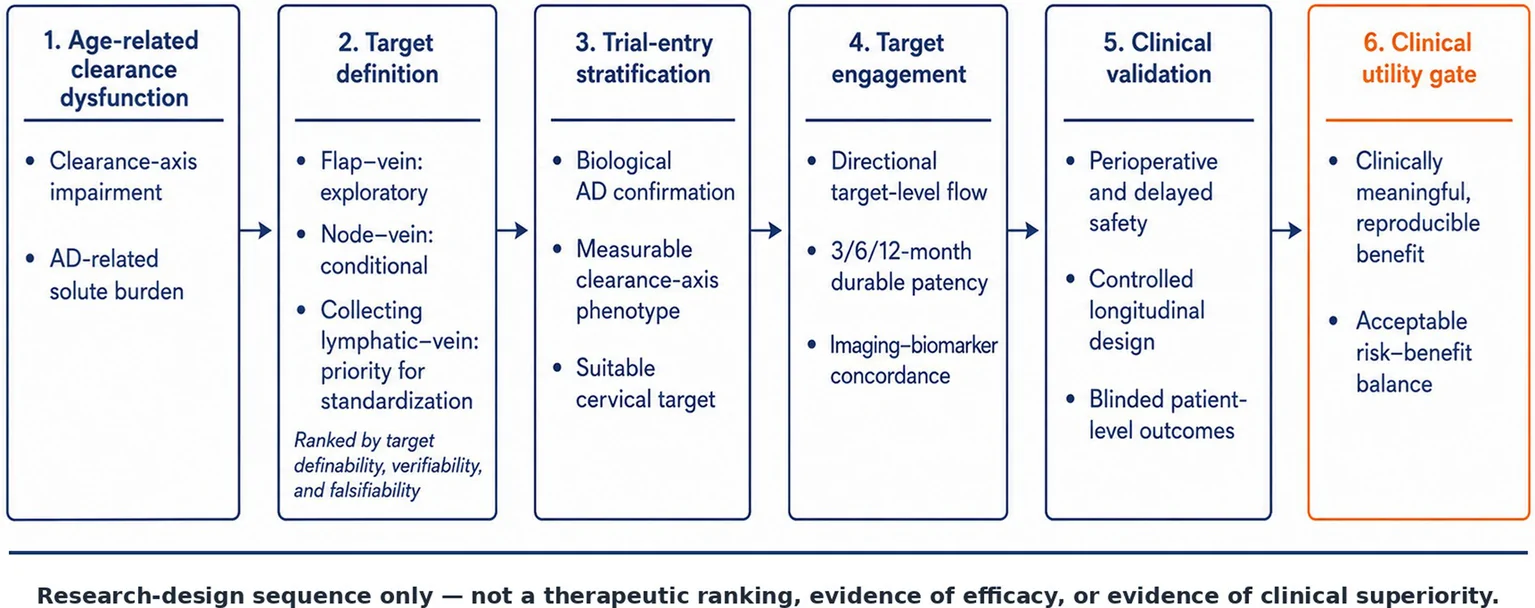
Conditional validation pathway for deep cervical lymphovenous reconstruction in Alzheimer’s disease. The framework moves from age-related clearance dysfunction through target definition and trial-entry stratification to target engagement, controlled clinical validation, and a clinical utility gate. Trial entry requires biological confirmation of AD, a measurable clearance-axis phenotype, and a suitable cervical target. Target engagement requires directional target-level flow, durable patency at 3, 6, and 12 months, and concordant imaging and biomarker findings. Clinical validation requires perioperative and delayed safety surveillance, a controlled longitudinal design, and blinded patient-level outcomes. Flap-vein reconstruction remains exploratory, node-vein reconstruction is conditional on demonstrated nodal function, and collecting lymphatic vessel-vein reconstruction is prioritized as the most directly definable and testable validation construct; this is not a therapeutic ranking or evidence of clinical superiority. Progression should occur only when prespecified go/no-go criteria are met. Clinical utility requires clinically meaningful, reproducible benefit and an acceptable risk–benefit balance. Feasibility does not establish efficacy; local patency does not establish disease modification; biomarker change does not establish clinical utility.

Non-surgical interventions also alter components of the glymphatic-lymphatic pathway. In 5XFAD mice, multisensory 40-Hz stimulation increased CSF influx and interstitial efflux, improved perivascular AQP4 polarization, enlarged meningeal lymphatic vessels, and reduced Aβ; blocking glymphatic clearance attenuated the Aβ effect, although neuronal and immune mechanisms may also contribute (Murdock et al., 2024). Four weeks of 808-nm photobiomodulation improved meningeal lymphatic drainage, pathology, and behavior in aged and AD-model mice, and lymphatic ablation reduced the benefit, supporting pathway dependence but not human efficacy (Wang M. et al., 2024). In humans, a 12-week exercise study found changes in putative glymphatic inflow and meningeal lymphatic MRI measures in 16 long-term trainees, whereas outflow measures and a 21-participant acute-exercise group did not show the same pattern (Yoo et al., 2025). Together, they support pathway modifiability and position cervical surgery as one experimental component of a broader clearance system, without establishing human AD efficacy (Zhang et al., 2026).

Other AD interventions provide useful methodological comparators even though their mechanisms lie outside the three-interface classification. In a 42-patient randomized sham-controlled fornix deep-brain-stimulation trial, Lozano et al. (2016) found no overall advantage on the primary 13-item Alzheimer’s Disease Assessment Scale-Cognitive Subscale (ADAS-Cog13) or CDR-SB outcomes despite a transient metabolic signal; a *post hoc* age interaction remained hypothesis-generating. An open 10-patient vagus-nerve-stimulation pilot reported small cognitive changes without sham control (Sjogren et al., 2002). In five patients, Lipsman et al. (2018) showed reversible magnetic resonance-guided blood–brain-barrier opening without a group-level amyloid reduction; in three patients, Rezai et al. (2024) reported approximately 32% greater regional amyloid-PET reduction in ultrasound-treated regions during aducanumab therapy, still without evidence of clinical benefit. A 29-patient randomized pilot of low-flow CSF drainage did not establish robust cognitive efficacy (Silverberg et al., 2002), and historical omental transposition relied on uncontrolled case observations (Goldsmith, 1996). Across these examples, technical or biological target engagement did not substitute for durable patient-level benefit.

Reporting, trial entry, and endpoint selection all require standardization. Operative reports must identify the verified lymphatic target, its upstream source, the recipient vein, anastomotic geometry, reflux-control strategy, operator experience, and local patency method. For collecting-vessel procedures, reports should also describe the vessel’s relation to adjacent nodes when this can be established, using the node-relative terminology defined in Section 4.3. Trial entry should distinguish pathology confirmation from clearance phenotyping as specified in Section 6.1: plasma or CSF p-tau217 can support the former, whereas DTI-ALPS, PVS burden, sleep and vascular physiology, cervical imaging, and the exploratory NPTX2/IGSF10 panel remain unvalidated candidate phenotypes for the latter (Therriault et al., 2023; Ashton et al., 2024; Huang et al., 2024; Li Y. et al., 2024; Kim et al., 2026; Li Y. et al., 2026). Endpoints should move from target-level directional patency to central and cervical imaging, then to process-specific fluid biomarkers and blinded patient-level outcomes. GFAP and p-tau181 add astroglial and tau-pathology information, but their diagnostic associations do not validate short-term change as a clinical surrogate (Yang et al., 2023).

Perioperative physiology and longitudinal sampling also need prespecified control. In anesthetized rodents, side-lying posture produced greater glymphatic transport than prone positioning, but this cannot be translated directly into a human treatment posture (Lee et al., 2015). Using 7-T real-time flow MRI in 10 healthy volunteers, Töger et al. (2022) showed rapid bidirectional CSF-flow changes during respiratory maneuvers; the method resolved respiratory effects but underestimated faster cardiac oscillations and did not measure net solute transport. Anesthesia type and depth, ventilation and carbon dioxide, blood pressure, fluids, inflammation, sleep, and surgical stress can all confound glymphatic measurements and short-term cognition (Dong et al., 2024). CLEAN-AD studies will be informative only if these factors are synchronized, the operative target is disclosed, and months-long directional patency is linked to central imaging, downstream cervical assessment, fluid biomarkers, and blinded outcomes. Cross-disease brain-lymphatic models can guide measurement but cannot import traumatic-brain-injury efficacy assumptions into AD (Dong et al., 2025).

Table 4 aligns candidate endpoints with the claims they can and cannot support.

**Table 4:** Endpoint hierarchy for aging-neuroscience studies.

Endpoint layer	Candidate indicators	Main value	Main limitation	Interpretive status
Anatomic/local patency	Intraoperative ICG plus repeated postoperative directional imaging at 3, 6, and 12 months.	Distinguishes immediate technical flow from durable target engagement.	ICG and near-infrared methods may miss deep targets; visible movement may be bidirectional or non-quantitative.	Necessary but not sufficient for any mechanism or efficacy inference.
Clearance-axis imaging	DTI-ALPS, PVS burden, MR lymphatic imaging, and Aβ/tau PET when indicated.	Tests whether central exchange and downstream anatomy change together.	DTI-ALPS and related metrics are indirect and sensitive to white-matter anatomy, vascular disease, motion, and protocol.	Mediator or stratification layer, not a clinical substitute.
Fluid biomarkers	p-tau217 for AD confirmation; GFAP/p-tau181 for glial/tau dimensions; serial Aβ42/40 and CSF Aβ/tau measures; exploratory NPTX2/IGSF10 response panel.	Separates pathology confirmation from exploratory redistribution and response hypotheses.	Diagnostic accuracy does not validate short-term treatment-response thresholds; peripheral sources and handling confound interpretation. The NPTX2/IGSF10 model is internally derived and lacks external validation, an untreated comparator, and target-specific patency linkage.	Biological-response layer interpreted only with anatomy, directional patency, imaging, and blinded outcomes.
Cervical downstream phenotype	Node morphology/perfusion; source- and direction-defined collecting-vessel mapping; selective aspiration where justified; recipient-vein pressure/reflux testing; living-human confirmation of cadaver-defined level II targets.	Tests target suitability, mixed tissue inputs, nodal processing, directional flow, and recipient-vein compatibility.	No validated AD action thresholds; invasive sampling, clustering, and operator dependence remain concerns. Cadaver ICG defines anatomy but not active in vivo flow or a surgically rate-limiting pathway.	Exploratory target-selection and mechanism layer.
Research-only eligibility gate	All four domains present: biomarker-confirmed AD; reproducible clearance-axis phenotype; interface-specific cervical target with defined source and directional flow; recipient-vein compatibility plus capacity for blinded longitudinal follow-up.	Converts selection concepts into a prespecified go/no-go screen and matches target evidence to the proposed interface.	No validated AD-specific thresholds; missing or discordant domains should defer enrollment rather than be imputed or offset by an isolated favorable result.	Protocol-level eligibility framework for prospective research, not a clinical recommendation.
Clinical and safety	CDR-SB, ADL, NPI, MMSE beyond measurement error, caregiver burden, quality of life, hemorrhage, thrombosis/reflux, serious or recurrent infection, viral reactivation, autoimmune or immune-mediated neurological events, persistent inflammatory or hematologic abnormalities, systemic vascular events, organ dysfunction, reintervention, and long-term harms, assessed at baseline and 3, 6, and 12 months with longer follow-up where feasible.	Captures patient-important benefit and local, neurological, systemic immunological, infectious, hematologic, vascular, and organ-level risk.	Requires adequate sample size, controlled comparators, blinded and independent event adjudication, active surveillance, clinically interpretable laboratory measures, and complete long-term follow-up.	Core utility and long-term safety layer for validation trials.
Axis-coupling trajectory	Paired central imaging, cervical flow/pressure, process-specific biomarkers, sleep/vascular physiology, and patient/caregiver outcomes.	Tests whether local target engagement propagates through the distributed clearance-inflammatory system.	Complex mediation, timing, and cross-center harmonization; cross-disease frameworks require AD-specific validation.	Mechanistic add-on for staged trials and registries.

Candidate thresholds and effect sizes require platform-specific validation before comparison. AD, Alzheimer’s disease; ADL, activities of daily living; Aβ, amyloid beta; CDR-SB, Clinical Dementia Rating-Sum of Boxes; CSF, cerebrospinal fluid; DTI-ALPS, diffusion tensor image analysis along the perivascular space; GFAP, glial fibrillary acidic protein; ICG, indocyanine green; IGSF10, immunoglobulin superfamily member 10; MMSE, Mini-Mental State Examination; MR, magnetic resonance; NPI, Neuropsychiatric Inventory; NPTX2, neuronal pentraxin-2; PET, positron emission tomography; p-tau, phosphorylated tau (numeric suffix denotes the residue); PVS, perivascular space.

### 6.5 Controversies and falsifiable predictions

The brain-meningeal-cervical pathway is distributed and redundant rather than pipe-like: skull-base, cribriform, cranial-nerve, nasopharyngeal, deep-cervical, and superficial-cervical routes may contribute differently with age and physiological state, and collateral cervical pathways can redirect flow after local perturbation (Ahn et al., 2019; Murakami et al., 1994; Papadopoulos et al., 2025; Yoon et al., 2024). Local patency does not establish net cerebral clearance, and nodal visibility or biomarker enrichment cannot define nodal function. Accordingly, preference for a collecting-vessel validation construct is methodological rather than therapeutic.

The updated evidence is directionally mixed. The APP/PS1 model of Liu et al. (2026) supports short-term tracer target engagement after node-EJV anastomosis, whereas the retrospective PET comparison of Jin L. et al. (2026) reported less favorable six-month imaging and cognitive trajectories after dcLVA than after lecanemab. Wang G. et al. (2026) further emphasized pressure disparity, anatomical uncertainty, lack of randomization, biomarker validation, and anesthesia control. None of these sources is definitive: the mouse model is not human efficacy evidence, the clinical comparison is non-randomized and interface-limited, and the Perspective is an analytical critique rather than an outcome study. Together they make the hypothesis more testable while arguing against a one-directional efficacy narrative.

The hypothesis would be weakened by failure to demonstrate durable target-level flow, by a lack of coupling between patency and clearance measures, or by unchanged blinded clinical outcomes despite biological engagement. Reproducible concordance across anatomy, flow, biomarkers, imaging, and patient outcomes would justify progression to later-stage trials.

## 7 Discussion

This review differs from previous general appraisals of dcLVA (Chen Q. et al., 2025; Li G. et al., 2026) by separating the lymphatic-side target. Flap-vein, node-vein, and collecting vessel-vein procedures reconstruct different structures and therefore require different evidence of flow, durability, and biological engagement. Primary classification is based on the structure directly connected to the vein; for collecting-vessel procedures, local relations to individual nodes are reported separately when known rather than used to create additional subtypes. The brain-meningeal-cervical axis is biologically plausible, but plausibility alone is not treatment evidence.

The rationale rests on aging biology rather than operative novelty. Age-related changes in sleep and circadian timing, vascular pulsatility, AQP4 organization, meningeal lymphatic integrity, nasopharyngeal input, and cervical nodal phenotype may jointly reduce clearance reserve (Da Mesquita et al., 2018; Hablitz et al., 2020; Kim et al., 2026; Yoon et al., 2024). These changes occur at different compartments and need not respond symmetrically to a distal bypass. Cervical reconstruction therefore perturbs one component of a distributed aging system rather than correcting AD pathogenesis as a whole.

Current AD reports are heterogeneous in target, method, disclosure, and outcome interpretation. Table 2 now includes feasibility cohorts, a dynamic vein-graft case series, a pilot stratification study, an exploratory proteomic study, a retrospective PET comparison, a delayed safety case, and registered studies. The mouse model of Liu et al. (2026) and cadaver mapping of Nguyen et al. (2026) expand mechanistic and anatomical testability without establishing human benefit. Li Y. et al. (2026) provide candidate response stratification without external validation; Chen et al. (2026) provide short-term technical observations from five patients; Jin L. et al. (2026) provide a non-randomized comparative signal that does not favor dcLVA; and Jiang et al. (2026) provide an important delayed safety observation without causal proof. No single report supplies target definition, durable directional patency, controlled biological engagement, and blinded patient-level benefit together. Each signal must therefore be interpreted according to the reconstructed target and its evidentiary level rather than under a generic dcLVA label.

Interface-specific validation requirements are summarized in Table 1 and Figure 5 and guide protocol design rather than treatment selection.

Clinical and biomarker signals remain worth testing, but short-term changes in uncontrolled studies can arise from measurement error, practice, caregiver expectation, perioperative care, sleep, inflammation, regression to the mean, or disease heterogeneity. Comparator fields show why a mechanistic signal cannot substitute for patient benefit: randomized fornix stimulation changed metabolism without improving the overall primary clinical outcomes (Lozano et al., 2016); focused ultrasound achieved reversible barrier opening or regional amyloid-PET change without establishing cognitive efficacy (Lipsman et al., 2018; Rezai et al., 2024); and randomized pilot CSF drainage did not establish robust benefit despite a plausible clearance rationale (Silverberg et al., 2002). The appropriate next step is a linked test of target anatomy, durable directional flow, upstream and downstream clearance measures, biomarker trajectories, delayed safety, and blinded patient-level outcomes—not simply a larger uncontrolled cohort.

The 2025 National Health Commission notice confines the procedure to approved research because indications and contraindications remain unclear and high-quality safety and efficacy evidence is insufficient. It requires adequate relevant preclinical evidence and ethics-committee deliberation before clinical research (National Health Commission of the People’s Republic of China, 2025). This position reflects a broader problem in surgical innovation: feasibility and biological plausibility can advance faster than standardized evidence. Current studies therefore require rigorous protocols, ethical oversight, complete adverse-event reporting, and staged validation rather than clinical promotion.

## 8 Limitations

This narrative review was not preregistered, and no formal risk-of-bias instrument, GRADE assessment, or quantitative synthesis was applied. The original multi-database search covered records through 15 July 2026; a targeted PubMed update, exact-phrase searches, publisher-record checks, and citation tracking were completed through 22 August 2026. Web of Science and Scopus were not re-run during revision, and recently published or incompletely indexed reports may still have been missed. The proposed ordering should be read as a falsifiable research hypothesis rather than an evidence grade.

Evidence quality is uneven. Foundational and human imaging studies support the existence and measurability of the brain-meningeal-cervical axis, whereas operative evidence is largely uncontrolled and short term. Several inferences about LNVA combine AD-specific nodal observations with classic or peripheral pressure-flow data. This combination challenges the assumption of a universally normal, low-resistance target node but does not directly measure pressure-flow behavior in AD cervical nodes. Medium-term durability concerns likewise derive mainly from animal and peripheral lymphatic surgery and do not establish inevitable failure of modern cervical reconstruction.

The lymphatic-side object cannot always be classified confidently because source reports omit operative detail, and the mapping in Table 2 cannot substitute for complete protocols or target-level flow verification. Even when a collecting vessel can be identified, available reports may not establish its source, direction, or local relation to individual nodes, which limits inference about the nodal processing retained or bypassed. Hu et al. (2026) add biomarker information without causal attribution or interface certainty. Jiang et al. (2026) add an important delayed safety observation, but a single temporally associated event with incomplete surgical detail cannot estimate incidence or establish mechanism. The review also focuses on structural cervical exit reconstruction and does not compare all molecular, physical, sleep, exercise, or neuromodulatory approaches to brain clearance.

Cross-disease and peripheral evidence is used to clarify mechanism and study design, not to transfer efficacy assumptions from traumatic brain injury or lymphedema to AD. All proposed imaging, biomarker, and safety measures require disease-specific, multicenter validation.

## 9 Conclusion

Deep cervical lymphovenous reconstruction in Alzheimer’s disease is not a single operation. Flap-vein, node-vein, and collecting vessel-vein interfaces differ in the reconstructed structure, pressure-flow behavior, likely failure modes, and capacity for objective verification. Recent mouse, cadaveric, proteomic, dynamic-graft, and retrospective comparative reports broaden mechanistic and translational signals, but human evidence remains largely uncontrolled, incompletely reported, and insufficient to establish a surgically rate-limiting cervical bottleneck or clinical efficacy.

For aging-neuroscience research, collecting lymphatic vessel-vein anastomosis is the most directly testable construct, LNVA is conditional on demonstrated nodal function, and lymphatic flap-vein reconstruction remains exploratory. This is a research-design hierarchy, not a therapeutic ranking. On current evidence, all three remain experimental and should be evaluated only in rigorously governed studies that establish biological AD, a targetable downstream phenotype, durable directional patency, concordant imaging and biomarker changes, delayed safety, and blinded patient-level benefit. No interface has established clinical efficacy or superiority in AD.

## Glossary

AD: Alzheimer’s Disease
ADAS-Cog13: 13-Item Alzheimer’s Disease Assessment Scale-Cognitive Subscale
ADL: Activities of Daily Living
Aβ: Amyloid Beta
APP/PS1: Amyloid Precursor Protein/Presenilin-1
AQP4: Aquaporin-4
AUC: Area under the Receiver Operating Characteristic Curve
CDR: Clinical Dementia Rating
CDR-SB: Clinical Dementia Rating-Sum of Boxes
CIBIC-plus: Clinician’s Interview-Based Impression of Change Plus Caregiver Input
CLEAN-AD: Deep Cervical Lymphatic Venous Anastomosis in the Treatment of Alzheimer’s Disease
CNS: Central Nervous System
CONSORT-NPT: Consolidated Standards of Reporting Trials extension for nonpharmacologic treatments
CSF: Cerebrospinal Fluid
dcLVA: Deep Cervical Lymphaticovenous Anastomosis
DTI-ALPS: Diffusion Tensor Image Analysis along the Perivascular Space
EJV: External Jugular Vein
FDG-PET: Fluorodeoxyglucose Positron Emission Tomography
GFAP: Glial Fibrillary Acidic Protein
GRADE: Grading of Recommendations Assessment Development and Evaluation
ICG: Indocyanine Green
IDEAL: Idea Development Exploration Assessment and Long-term study
IGSF10: Immunoglobulin superfamily member 10
ISF: Interstitial Fluid
LNVA: Lymph Node-Vein Anastomosis
LVA: Lymphaticovenous anastomosis
MMSE: Mini-Mental State Examination
MoCA: Montreal Cognitive Assessment
MRI: Magnetic Resonance Imaging
NPI: Neuropsychiatric Inventory
NPTX2: Neuronal pentraxin-2
PET: Positron Emission Tomography
PROBE: Prospective Randomized Open Blinded-Endpoint
p-tau: Phosphorylated Tau Numeric Suffix Denotes the Phosphorylation Residue
PVS: Perivascular Space
STROCSS: Strengthening the Reporting of Cohort Cross-sectional and Case–control Studies in Surgery
SUVR: Standardized Uptake Value Ratio
VEGF-C: Vascular Endothelial Growth Factor C
